# Centrality of drug targets in protein networks

**DOI:** 10.1186/s12859-021-04342-x

**Published:** 2021-10-29

**Authors:** Ariele Viacava Follis

**Affiliations:** EMD Serono Research and Development Inc., 45A Middlesex Turnpike, Billerica, MA 01821 USA

**Keywords:** Drug target, Protein network, Graph analysis

## Abstract

**Background:**

In the pharmaceutical industry, competing for few validated drug targets there is a drive to identify new ways of therapeutic intervention. Here, we attempted to define guidelines to evaluate a target’s ‘fitness’ based on its node characteristics within annotated protein functional networks to complement contingent therapeutic hypotheses.

**Results:**

We observed that targets of approved, selective small molecule drugs exhibit high node centrality within protein networks relative to a broader set of investigational targets spanning various development stages. Targets of approved drugs also exhibit higher centrality than other proteins within their respective functional class. These findings expand on previous reports of drug targets’ network centrality by suggesting some centrality metrics such as low topological coefficient as inherent characteristics of a ‘good’ target, relative to other exploratory targets and regardless of its functional class. These centrality metrics could thus be indicators of an individual protein’s ‘fitness’ as potential drug target. Correlations between protein nodes’ network centrality and number of associated publications underscored the possibility of knowledge bias as an inherent limitation to such predictions.

**Conclusions:**

Despite some entanglement with knowledge bias, like structure-oriented ‘druggability’ assessments of new protein targets, centrality metrics could assist early pharmaceutical discovery teams in evaluating potential targets with limited experimental proof of concept and help allocate resources for an effective drug discovery pipeline.

**Supplementary Information:**

The online version contains supplementary material available at 10.1186/s12859-021-04342-x.

## Background

Pharmaceutical companies strive to select suitable targets and minimize attrition. This has driven more than two decades long efforts towards the identification and annotation of ‘druggable’ fractions of the genome [1–3]. A seminal study by Hopkins et al. evaluated proteins’ domain composition combined with their role in disease [2], proposing a ‘druggable’ subset of the proteome comprised of 600–1500 proteins. The analysis of biological signaling and/or protein interaction networks has provided an appealing orthogonal approach to the identification of potential drug target genes [4–12].

Many naturally existing networks, including biological signaling networks, exhibit an approximate [13] scale-free organization characterized by a power law dependence of their node degree distribution [14–17]. In scale-free networks few hub nodes exhibit high connectivity relative to most nodes, as opposed to a normal node degree distribution observed in random networks. Scale-free organization results in short across-network distances and confers a network robustness to the perturbation of a limited number of its edges [18]. These characteristics are intuitively advantageous to biological signaling as they help fulfill the conflicting requirements of efficient response to external stimuli (short distance) while preserving homeostasis upon perturbation (robustness) [15]. Although each protein (node) has a specific function, hub proteins in signaling networks may play gateway roles at a higher hierarchical level [19].

The application of graph theory to the analysis of biological networks has been largely focused on the ‘architecture’ of biological signaling [20, 21]. Some studies investigated the signaling context of drug targets in network models to identify potential toxicity liabilities [22], drugs repurposing [23] or polypharmacology [24]. Others, closer to the scope of the current work, applied diverse analytical methods to a range of annotated networks with the general goal of investigating node characteristics that may discriminate specific drug targets from other proteins [4–12]. Some of these studies focused on distinctive features of drug target nodes [4, 8–10, 12]; others analyzed bipartite networks (composed of two separate sets of nodes that connect with each other [25]) of drugs and drug targets [5–7, 11].

These studies highlighted a few general trends and some contradictions. Depending on the analysis and networks [26–31] it was applied to, varying discriminants in network characteristics were detected between drug targets and other proteins. These ranged from rather complex local network features of drug target nodes [4, 11, 12] to simpler node centrality metrics (in primis node degree) [6, 8], with drug targets in general exhibiting higher centrality than other proteins. These results extensively demonstrated that certain classes of proteins are likelier drug targets than others yet featured limited investigation of which proteins within each class may be likelier drug targets [8, 12]. We recognized that the network centrality features detected for drug targets may be influenced by their biased protein class distribution relative to other proteins [3, 7] as proteins belonging to different functional classes may exhibit inherently different positioning and centrality metrics within network models.

As annotated biological signaling or protein interaction networks are influenced by their underlying data sources, annotation method, and completeness, so may be the outcome of analyses applied to these networks. Recognition of this possible source of bias through cross comparison of different networks or generation of consensus networks was limited in previous studies [8, 12].

In the current study, we attempted to address discrepancies apparent from the comparison of previous studies and implement a broad evaluation of node centrality metrics along with parallel comparison of multiple source networks/databases. We reasoned that such comparative inspection would minimize any bias derived from their different annotation sources and assembly strategies. In our analysis we evaluated whether any network positioning and centrality features would discriminate ‘ideal’ target proteins, associated to selective marketed drugs not only from the entire proteome, but also from other proteins of potential pharmaceutical interest. Additionally, we dissected comparisons between network characteristics of drug targets versus other proteins over their respective target classes to identify differences that would not merely arise from the biased target class composition of drug targets. Last, we evaluated the entanglement between protein nodes characteristics within annotated functional networks and their literature enrichment as a measure of the knowledge bias that may influence the outcome of these analyses.

## Results

### Datasets selection and annotation

**Fig. 1:**
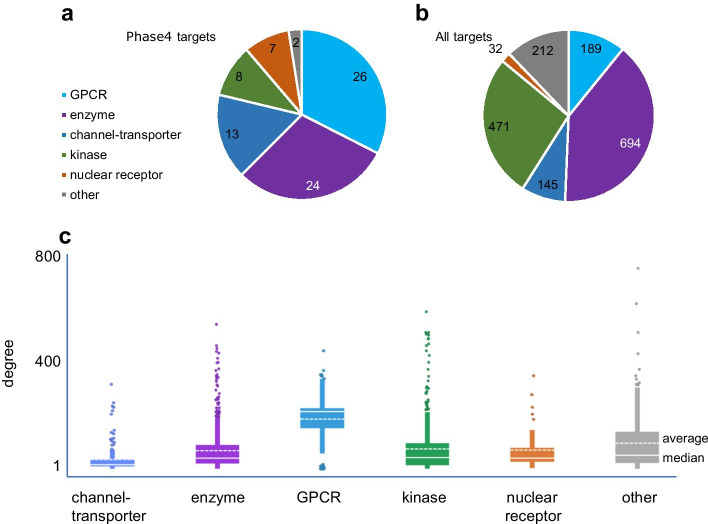
Datasets selection. Target class composition of Phase4 targets (**a**) and all targets (**b**) datasets. **c** Different degree distribution between target classes in the String network mapped at edge confidence level = 0.7

Previous studies demonstrated that drug target proteins in general exhibit higher centrality within signaling networks than other proteins [4–12]. To investigate this finding in more depth, here we identified a subset of proteins targeted by marketed, highly selective drugs (defined as ‘Phase4 targets’) and compared them to the complete set of exploratory or discovery targets (defined as ‘all targets’). These two sets were identified within the ChEMBL database [32] (version 27, 2020) respectively as individual protein targets of approved drugs reported to interact with four or less proteins (Phase4 targets, 80 proteins) and as the entire set of proteins with at least 40 reported interacting small molecules, regardless of the compounds development stage (all targets, 1743 proteins). Only individual protein targets were considered, as targets annotated as protein families or complexes (i.e. composed of multiple nodes) would convolute the analysis. Proteins within each set were assigned to a broad ‘target class’ based on Gene Ontology (GO) [33] identifiers: channels and transporters, enzymes (excluding kinases), G-protein coupled receptors (GPCRs), kinases, nuclear receptors (Fig. 1a, b). Targets that did not belong to any of these classes were classified as ‘other’. Target classes were deliberately broad to ensure that each class would be sufficiently populated to allow a statistical evaluation of differences between their graph node parameters. The Phase4 targets set is limited, and by excluding targets of less selective compounds, it does not include all the targets of approved drugs, yet it represents a comprehensive spectrum of therapeutic areas (Additional file 1: Fig. S1). We focused on targets of selective drugs to avoid convolution with potential poly pharmacology effects and strive to identify properties of effective individual protein targets.

### Centrality analysis of target nodes in a protein signaling network

We first analyzed these sets of proteins by calculating their node parameters within the String database network [31, 34] (version 11.0, human proteins) mapped at a confidence cutoff of 0.7 (‘high’ confidence—‘String0.7’). The resulting network contained 17,161 nodes (proteins) and 419,761 undirected edges. String is a meta-database sourced from most publicly available curated databases of protein interactions or protein functional connections, large datasets and automated keyword mining. String edges are thus broad descriptors of associations between proteins, not limited to physical interactions. Edges from un-curated sources are assigned a confidence score based on an estimated likelihood of randomly identifying an association between two proteins (false positives). We chose String for this initial analysis specifically because of its broad, inclusive method of annotation which would minimize gaps in the network, at the cost of potentially including incorrectly assigned edges (depending largely on the choice of confidence level cutoff; how this was addressed will be discussed later on). We calculated standard centrality metrics [35, 36] measuring the extent of connections and local network characteristics for all the nodes in this network. Table1 includes a brief description of each studied parameter, divided between scalar properties that depend on the network size versus normalized ones.

**Table 1:** Definitions of the centrality parameters considered in this analysis

*Size dependent parameters*
**Average shortest path**: average distance between an examined node and all other nodes
**Degree**: number of edges connected to an examined node
**Eccentricity**: largest number of edges between an examined node and any node in the network
**Neighborhood connectivity**: average number of edges of nodes neighboring an examined node
**Stress**: number of shortest paths between any two nodes passing through an examined node
*Normalized parameters*
**Betweenness centrality**: fraction of shortest paths between any two nodes passing through an examined node
**Closeness centrality**: normalized reciprocal distance between an examined node and any node
**Clustering coefficient (local)**: observed fraction of all possible edges between nodes neighboring an examined node
**Topological coefficient**: fraction of nodes neighboring an examined node that are shared with other nodes

Upon compilation of the Phase4 and all targets sets we observed a different distribution of target classes between the two sets (Fig. 1a, b). Additionally, the analysis of centrality metrics showed that several node parameters exhibited significantly different distribution ranges across different target classes (Fig. 1c, Additional file 1: Fig. S2, Table S1). These variations are likely related to the diverse broad functional contexts of each target class. Statistical differences between the *entire* Phase4 and all targets protein sets may depend in part on the different target class distribution between these two sets, however this bias is eliminated when comparing Phase4 and all target proteins within individual target classes. We therefore compared differences in centrality metrics between Phase4 and all targets within each target class (Table 2). These comparisons unavoidably attempt to interpret differences in complex underlying network structures through the simplified lens of statistical testing. Node centrality parameters in scale-free networks deviate from normality to varying extents (e.g., clustering coefficient exhibited a quasi-normal distribution in String0.7, but degree exhibited a long-tailed distribution—Additional file 1: Fig. S3). Non-parametric statistics are commonly used for the comparative analysis of these parameters [37, 38]. Conversely, there are literature precedents for the application of linear regression statistics to large non-normal samples based on the Central Limit Theorem [39, 40]. Furthermore, nodes exhibiting extreme values in centrality parameters are not merely outliers as these values reflect their true position within the network. We opted to evaluate statistical differences between Phase4 and all targets over both linear regression (sample means) and non-parametric rank ordering. We corrected these raw probabilities for multiple testing over the number of centrality parameters and target classes examined. The difference probabilities between Phase4 and all targets for the various centrality parameters exhibited general agreement between statistical approaches with large deviations limited to stress and betweenness centrality (Table 2, Additional file 1: Fig. S4).

**Table 2:**
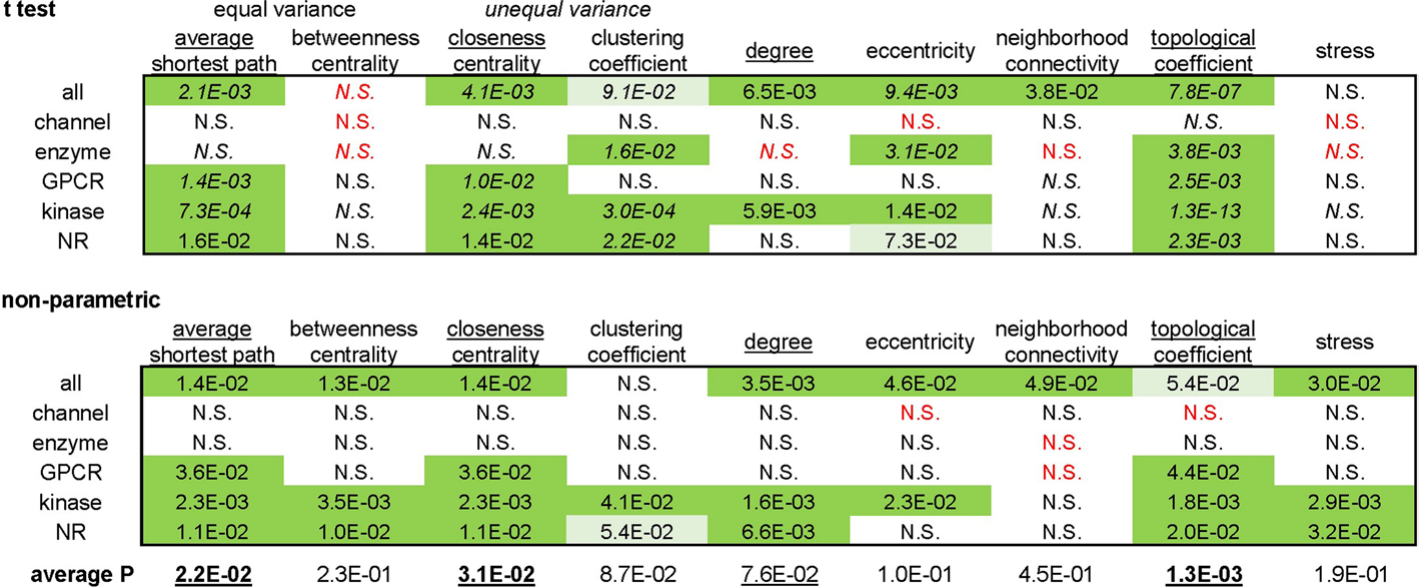
Null hypothesis probabilities for differences in centrality parameters between Phase4 targets and all targets, corrected for multiple testing across both centrality metric and target classes (Benjamini–Hochberg method)

Cells are coded according to statistical significance (uncorrected α = 0.05; dark green: significant, light green: marginal significance, N.S.: not significant). Red fonts indicate lower average (t-test) or median (non-parametric) centrality observed for Phase4 targets, regardless of significance. Average probabilities are calculated over all pairwise comparisons between Phase4 and all targets (‘all’ and class specific, t test and non-parametric)

**Fig. 2:**
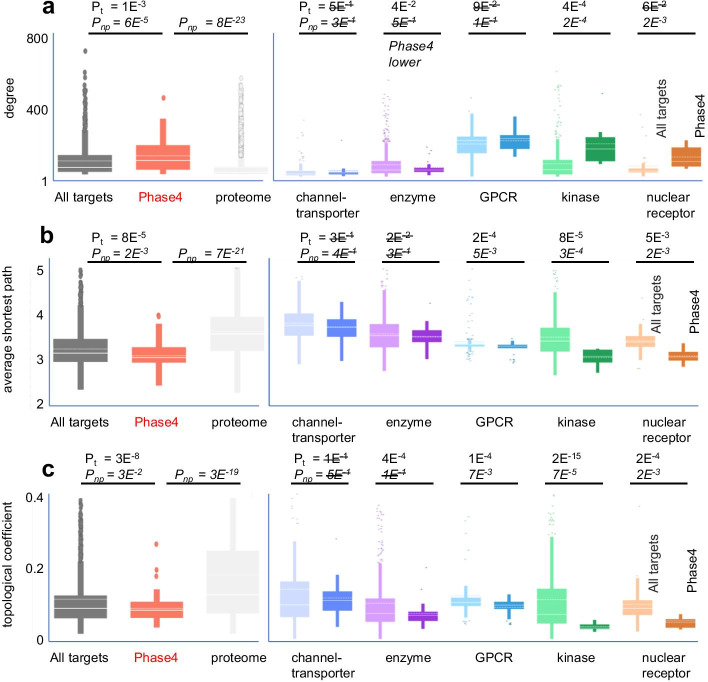
Different centrality metric distributions between Phase4 targets, all targets and proteome (left graphs) and between Phase4 targets and all targets across target classes (right graphs) identified from analysis of the String0.7 network. The plotted parameters are: degree (**a**), average shortest path (**b**), topological coefficient (**c**). Null hypothesis probabilities of differences between samples are marked as P_*t*_ for *t*-test and *P*_*np*_ for non-parametric testing, respectively. These values indicate raw pairwise probabilities and are not corrected for multiple testing as in Table 2. Values above significance threshold are crossed out

Several node parameters exhibited significantly different value ranges between Phase4 and all targets, according to either normality assumption or non-parametric testing, including a difference in degree as the simplest centrality metric (Table 2). In most class specific comparisons however Phase4 targets did not exhibit higher average or median degree than all targets (Fig. 2a). To identify which node parameters would better discriminate between Phase4 and all targets, we calculated for each parameter the average probability of increased centrality for Phase4 targets (*t*-test and non-parametric) across the entire dataset and class-specific comparisons (Table 2). Based on this assessment, the following two centrality metrics exhibited the largest differences between drug targets and all targets, retained in most class specific comparisons: drug targets exhibited *lower average shortest path* (Fig. 2b, equally its normalized reciprocal, higher closeness centrality) and *lower topological coefficient* (Fig. 2c). Pairwise comparisons of the extent of these parameters differences across target classes indicated some correlation between them, suggesting that they may be overall indicators of higher centrality of Phase4 targets relative to other target proteins (Additional file 1: Fig. S5).

**Fig. 3:**
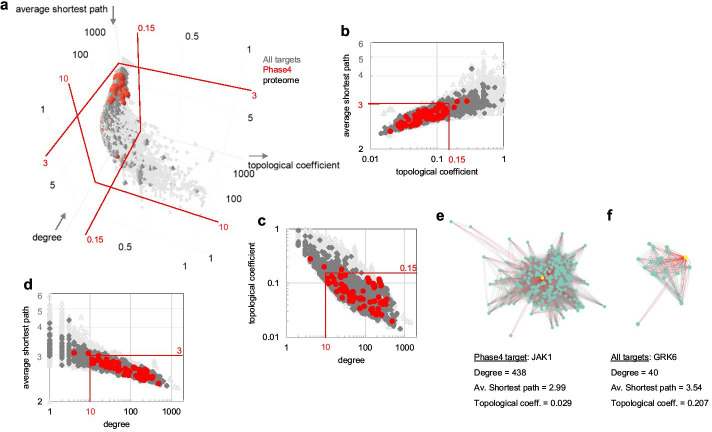
Scatter plots of centrality metrics distributions showing significant differences between Phase4 targets and all targets (**a–d**). Three-dimensional plot of average shortest path vs. degree vs. topological coefficient (**a**), and pairwise two-dimensional projections with logarithmic scaling (**b–d**). Red lines and values indicate approximate parameter boundaries enclosing most Phase4 targets. **e** and **f** Examples of local network connectivity of Phase4 and all targets kinases with different degrees, average shortest path and topological coefficient. The Phase4 kinase JAK1 is more central within its local network than GRK6

A low topological coefficient may provide a simplified, approximate descriptor of ‘good’ drug targets. The lower than average value of this parameter for Phase4 targets relative to all targets was observed across most target classes and it is not merely an artifact caused by differential class distribution between Phase4 and all targets. Additionally, this parameter exhibits limited variation between target classes, simplifying the assignment of a single cutoff value indicative of a target’s fitness independent of its target class (Fig. 2c, Additional file 1: Table S1). For the current String0.7 network analysis this value would be approximately 0.15 (contingency Chi square *P* < 10^−4^, Fig. 3a–c). More meaningful distinctions between Phase4 and all targets however may be identified through combination of values for multiple centrality parameters (e.g. Figure 3a–d), possibly with target-class specific patterns, as will be discussed later. A general interpretation of this finding is that ‘good’ targets may be gateway proteins (low distance, high degree) to self-standing signaling networks (low topological coefficient). Modulating the function of such proteins may be less susceptible to network robustness, which could enable compensation of a drug effects through redundant or overlapping signaling mechanisms (Fig. 3e–f).

### Assessing knowledge bias in node centrality metrics

We sought to address the critical prospect that this result could be a *post factum* consequence of the circumstance that drug targets are extensively studied proteins. Such knowledge bias could inherently impart higher centrality measures to drug target nodes in annotated networks derived from literature sources. In order to evaluate the possibility and extent of this convolution, we performed the following additional analyses: (1) we repeated the above described evaluation of node descriptors from additional networks generated through diverse annotation strategies; (2) we compared differences in centrality between Phase4 and all targets (subdivided in their respective target classes) against their relative number of literature references (within each network and across networks); (3) we compared dataset-wide correlations between centrality metrics of individual nodes and number of associated references to the probabilities of increased Phase4 targets centrality within each network.

First, we analyzed node centrality parameters for additional publicly available protein signaling networks (Table 3): String database at two additional confidence cutoffs (0.5 and 0.9, respectively lower and higher than the original analysis at 0.7 confidence–at 0.9 confidence String excludes any inferred, un-curated edges) hereby defined String0.5 and String0.9; BioGRID [28] (a curated database of experimentally determined genetic dependencies and protein–protein interactions from multiple sources); HumanNet XN [41] (a combined network of annotated functional associations and ortholog inferred associations); Reactome [42] (a curated network focused on mapping of signaling pathways); InBioMap [43] (a protein interactions network integrating multiple sources to aid the interpretation of large genomic datasets).

**Table 3:** General features of analyzed networks from annotated protein functional databases

	Nodes	Edges	Average degree	Coding genome coverage*
String 0.7	17,161	419,761	48.92	0.84
String 0.9	12,272	252,558	41.16	0.60
String 0.5	19,147	685,939	71.65	0.94
BioGRID	20,858	453,890	43.52	1.02
HumanNet	17,926	525,537	58.63	0.88
Reactome	14,071	268,857	38.21	0.69
InBioMap	17,653	625,641	70.88	0.87

^*^Full Uniprot, human, reviewed = 20,353

This assessment indicated that differences in centrality between Phase4 and all targets identified in the String0.7 network were not completely robust to switch of protein functional network sources, partially reconciling the conflicting conclusions of earlier studies [5–7, 12], they were however largely retained across different networks (Table 4). Higher centrality of Phase4 targets was more robust across databases for nuclear receptors, kinases and GPCRs than enzymes, channels-transporters. Phase4 channels and transporters exhibited either no difference or inverted (lower) trends in centrality relatively to comparable target proteins. Phase4 enzymes exhibited a lower average degree than other target enzymes in six of seven networks, but relatively low average topological coefficient in five of seven networks (significant in three). We have previously identified from analysis of the String0.7 network a low topological coefficient as a centrality indicator.

**Table 4:**
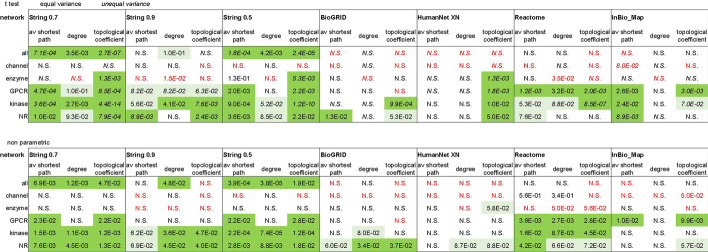
Null hypothesis probabilities for differences in centrality parameters between Phase4 targets and all targets tested in networks from different database sources, corrected for multiple testing across both centrality metric and target classes (Benjamini–Hochberg method)

Cells are coded according to statistical significance (uncorrected α = 0.05; dark green: significant, light green: marginal significance, N.S.: not significant). Red fonts indicate lower average (*t*-test) or median (non-parametric) centrality observed for Phase4 targets, regardless of significance

We measured differences between Phase4 and all targets after degree ‘normalization’ of the nodes’ topological coefficient [*log* (degree*topological coefficient), Table 5] and observed that this combined parameter was markedly lower (significantly or near significance) for Phase4 enzymes in networks where their degree but not their topological coefficient had been significantly lower than that of other enzymes (String0.9, Reactome), confirming that Phase4 enzymes broadly exhibit lower topological coefficient than other target enzymes relative to the extent of their connections after this degree ‘normalization’. The *log* (degree*topological coefficient) of Phase4 targets was higher than that of comparable targets for other target classes (kinases, nuclear receptors), indicating a higher degree as the dominant centrality metric for these target classes. This combined parameter exhibited generally minor differences between Phase4 and other GPCRs suggesting that the increased centrality of Phase4 GPCRs reflects with minimal deviation the reverse correlation between nodes degree and topological coefficient.

**Table 5:**
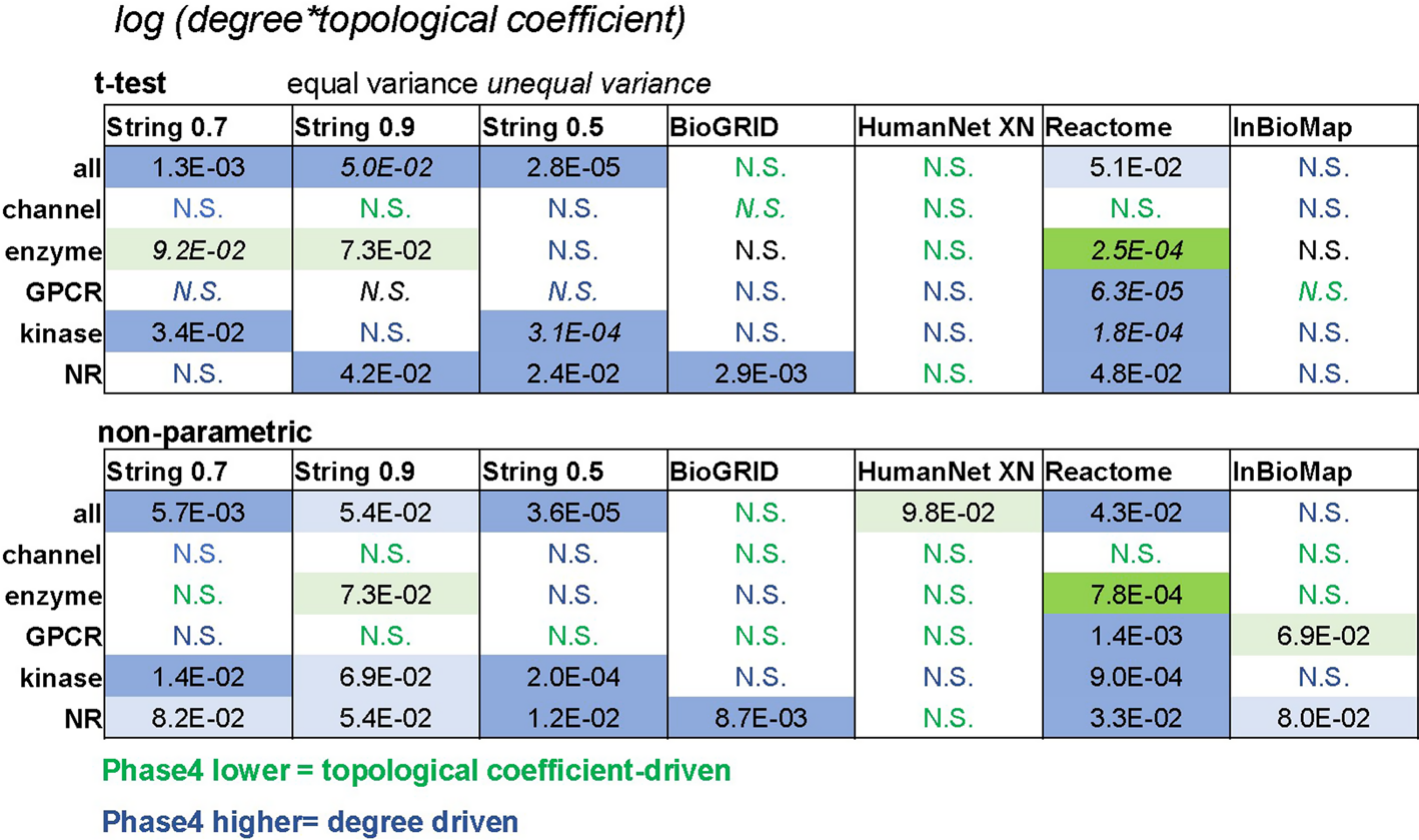
Null hypothesis probabilities for differences in the ‘degree-weighted’ parameter *log* (degree*topological coefficient) between Phase4 targets and all targets tested in networks from different database sources, corrected for multiple testing across target classes (Benjamini–Hochberg method)

Cells are color coded according to the relative difference between Phase4 and all targets. Green highlights lower values observed for Phase4 targets, indicating a dominant effect of differences in topological coefficient on the combined parameter. Blue highlights higher values observed for Phase4 targets, indicating a dominant effect of differences in degree on the combined parameter. Solid color boxes indicate statistically significant differences (uncorrected α = 0.05; dark shades: significant, light shades: marginal significance, N.S.: not significant)

### Comparisons between protein functional interaction networks

These analyses highlighted that differences in node parameters distributions between Phase4 and all targets depend on the different data sources and filters applied in the generation of each network. Trivially, these multiple annotation approaches result in varying proteome coverage within each network. We compared the networks’ size to the coding human genome as annotated in UniProt [44]—20,353 proteins (Table 3). The average proteome coverage over the seven networks was 83%, with String0.7, HumanNet XN and InBioMap falling close to this value; String0.5 (low confidence cutoff) and BioGRID exhibited a higher coverage while String0.9 (high confidence cutoff) and Reactome exhibited incomplete proteome coverage (< 70%). The overlap between analyzed networks was more marked for the Phase4 and all targets protein sets (Additional file 1: Tables S2, S3). These observations do not account for fluctuations in centrality metrics between networks of comparable sizes, that depend on the specific connectivity between nodes. Pairwise comparisons between the node parameters of target proteins sets across networks evidenced similarities and differences among them (Additional file 1: Table S4). Correlations varied across parameters and were intuitively higher between the three String networks mapped at difference edge confidence levels. Conversely, BioGRID and HumanNet XN exhibited low node parameter correlation to other networks, but generally good correlation with each other.

Differences between networks generated using different methods raise the critical question of how much is a network biased by its data sources relative to the ‘true’ underlying biological system it attempts to describe. It is obviously not possible to fully address this question as our entire knowledge of human biology (and anything else) is but a model or representation, we lack therefore a control system representing a ‘true’ network, neat of any knowledge bias. We define ‘exclusive’ and ‘inclusive’ forms of knowledge bias: by exclusive knowledge bias we mean the preferential incorporation of curated data sources with a likely deficiency in interactions (edges) that have not been expressly investigated through dedicated peer reviewed studies (e.g. Reactome, String0.9). By ‘inclusive’ knowledge bias we mean inclusion of inferred, not curated connections, some of which may be incorrectly assigned (e.g. String0.5, HumanNet XN). Less studied protein nodes would be inherently more susceptible to either form of bias than highly studied ones. We analyzed the networks’ node parameters distributions as one way to infer the extent and nature of their knowledge bias. A second key assessment consisted in evaluating relationships between centrality of protein nodes and extent of related literature.

### Differences in parameters distributions between networks

The tested networks exhibited scale-free character with cumulative node-degree distribution exponential between − 2 and − 3, minimal low degree saturation (fewer than expected low degree nodes) and no high degree cutoff (lower than expected maximum degree, Additional file 1: Fig. S6a) [17]. Low degree saturation was more marked in networks enriched in inferred edges (String0.5, HumanNet XN). This may be indicative of incorrectly assigned edges to low connectivity nodes—inclusive bias. Three networks (BioGRID, Reactome, InBioNet) had lower than 2 exponentials in their node-degree distribution fit to the 100–1000-degree range. Such anomalous node-degree distribution results from a disproportionate number of edges associated to high degree nodes. In the specific case of functional protein interaction networks this could be diagnostic of exclusive bias and preferential inclusion of curated data related to highly studied proteins. Subtler variations in the relative populations of different degree ranges were observed between networks, suggesting additional, more convoluted compositional biases in their structure (Additional file 1: Fig. S7).

We next inspected correlations between degree and clustering coefficient for the analyzed networks (Additional file 1: Fig. S6b). Relative to the other networks, BioGRID exhibited a marked hierarchical organization (low clustering coefficient at high degree), and low overall clustering. This could be another manifestation of the exclusive bias hypothesized from the node-degree distribution of this network. In conclusion, the diverse annotation strategy underlying the assembly of each protein network is reflected in variations in their overall architecture and organization. The exact nature of an underlying ‘true’ network remains elusive. Networks from curated sources are necessarily incomplete, while more inclusive networks may contain several incorrectly assigned edges. In the absence of a single faultless network, we deem as likely correct observations that are robust across most networks regardless of their specific variations.

### Influence of relative number of citations on node centrality metrics

We further assessed the extent of knowledge bias on networks composition, and consequent differences in node centrality between Phase4 and all targets, by evaluating relationships between number of citations and nodes centrality. We counted the number of literature references listed in PubMed (https://pubmed.ncbi.nlm.nih.gov) for each target (searched by their gene name abbreviations). We compared the relative abundance of Phase4 targets within each target class to the relative abundance of their citations and found that Phase4 targets have on average 2.5 fold more citations than all targets, with uneven distribution among target classes (ranging from a ratio of 0.5 for channels—transporters to ~ 13 for kinases, Table 6, Additional file 1: Fig. S8). Across databases, modest correlations were observed between the number of PubMed records and centrality metrics of each node with R^2^ values between 0.03 and 0.20 (Additional file 1: Fig. S9). Correlations were more marked in String0.5, followed by String0.7. This observation is in apparent conflict with the lower dependency of the String0.5 and String0.7 network structures on highly studied proteins with extensive curated data, due to the additional inclusion in these networks of inferred edges with confidence scores < 0.9. We hypothesize that text-mining, used among other algorithms to define inferred edges, could be sensitive to the recurrence of highly studied protein keywords. In this scenario, one of the nodes of several inferred edges would be a highly studied protein, potentially introducing some correlation between node centrality and number of literature references.

**Table 6:** Number of citations for Phase4 targets and all targets across target classes

	All citations	Phase4 citations	Fraction Phase4 citations	Relative abundance Phase4 citations in target class
All	1,781,808	208,785	0.12	2.5
Channel	65,138	2708	0.04	0.5
Enzyme	601,619	54,934	0.09	2.5
GPCR	59,071	6294	0.11	0.8
Kinase	471,280	107,867	0.23	13.1
NR	62,657	35,015	0.56	2.6

We compared the enrichment in citations of ‘Phase4’ targets relative to ‘all targets’ proteins to their difference in centrality *in each target class*, within individual networks and across networks. This analysis identified minimal association between class sorted Phase4 targets literature enrichment and their centrality, limited to the String0.5 network (Table 7, Additional file 1: Fig. S10a–b). We then compared correlations between node centrality parameters and number of literature references within each network to the centrality of Phase4 targets *across target classes*. This analysis revealed significant trends between the probability of Phase4 targets’ higher centrality in average shortest path; topological coefficient; degree and existing correlations between these parameter values and citations counts for individual nodes (Additional file 1: Figs. S9, S10c–e). As the combined parameter *log* (degree*topological coefficient) (Table 5) introduces reciprocity between two centrality measures, its correlations with the number of PubMed records were largely abrogated in all networks (Table 8, Additional file 1: Fig. S11). Significant differences in this parameter between Phase4 and all targets therefore ought to identify features of drug target nodes unbiased by their relative enrichment in literature citations. Cumulatively, these analyses evidenced some entanglement between network structures, extent of literature available for each of their component nodes, and outcome of any analyses performed on these networks. In this ‘chicken and egg’ situation, it is impossible to ultimately discriminate whether certain proteins are highly studied because they are true biological hubs, or they appear as hubs in annotated networks because they are highly studied proteins. We will limit ourselves to the observations that correlations between each protein node centrality metric and number of citations were modest in all the analyzed networks. Differences in centrality between Phase4 and all targets did not exhibit significant associations with their relative literature enrichment across networks when sorted by target class, supporting that literature enrichment of Phase4 targets alone is insufficient to justify these observations. It is also evident however that literature bias may contribute to the *extent* of increased Phase4 targets centrality in some of the analyzed networks (especially String0.5).

**Table 7:** Correlation between enrichment in citations and probability of differences in centrality metrics between Phase4 targets and all targets class (not corrected for multiple testing)

Network	String 0.7			String 0.9			String 0.5			Biogrid			HumanNet XN			Reactome			InBioMap		
	Shortest path	Degree	Topol. coeff	Shortest path	Degree	Topol. coeff	Shortest path	Degree	Topol. coeff	Shortest path	Degree	Topol. coeff	Shortest path	Degree	Topol. coeff	Shortest path	Degree	Topol. coeff	Shortest path	Degree	Topol. coeff
Adjusted R	0.30	0.30	0.50	− 0.02	− 0.09	− 0.05	**0.58**	0.38	**0.69**	− 0.11	0.34	0.54	− 0.06	0.15	0.004	− 0.24	− 0.23	0.06	− 0.20	0.26	− 0.16
P	0.15	0.15	0.07	0.40	0.49	0.43	**0.05**	0.11	**0.03**	0.51	0.13	0.06	0.46	0.24	0.37	0.90	0.81	0.65	0.69	0.17	0.61

Significant correlations are marked in bold (α = 0.05)

**Table 8:** Correlation between enrichment in citations and probability of differences in the ‘degree-normalized’ parameter *log* (degree*topological coefficient) between Phase4 targets and all targets in each target class (not corrected for multiple testing)

	log (degree*topological coefficient)						
Network	String 0.7	String 0.9	String 0.5	BioGRID	HumanNet XN	Reactome	InBioMap
Adjusted R	− 0.09	0.11	0.31	− 0.12	0.04	− 0.28	0.15
P	0.49	0.27	0.15	0.53	0.33	0.74	0.24

No significant correlation was observed

### Effect of network randomization on node centrality

We sought to evaluate the network structural features underlying the centrality metrics characteristic of Phase4 targets. We performed a series of network randomizations and analyzed their effect on the node centrality parameters identified as indicative of drug targets’ fitness. First, we performed a degree preserving randomization of the String0.7 network. In this randomization, edges are randomly rearranged while the original degree of each node is retained. In the resulting synthetic network, we observed that the differences in other centrality measures (average shortest path, topological coefficient) between Phase4 and all targets were largely retained across target classes (Table 9). Additionally, the fluctuation in these differences after randomization exhibited some correlation with the extent of (unchanged) differences in degree (Additional file 1: Fig. S12). This suggests that differences in average shortest path and topological coefficient between target sets are mostly projections of differences in degree, whose significance is amplified under given underlying network structures, independent of the specific connectivity of individual nodes. We hypothesized that such amplification of differences in shortest path and topological coefficient between Phase4 and all targets relative to their differences in degree may depend on the scale-free characteristics of the biological signaling network.

**Table 9:**
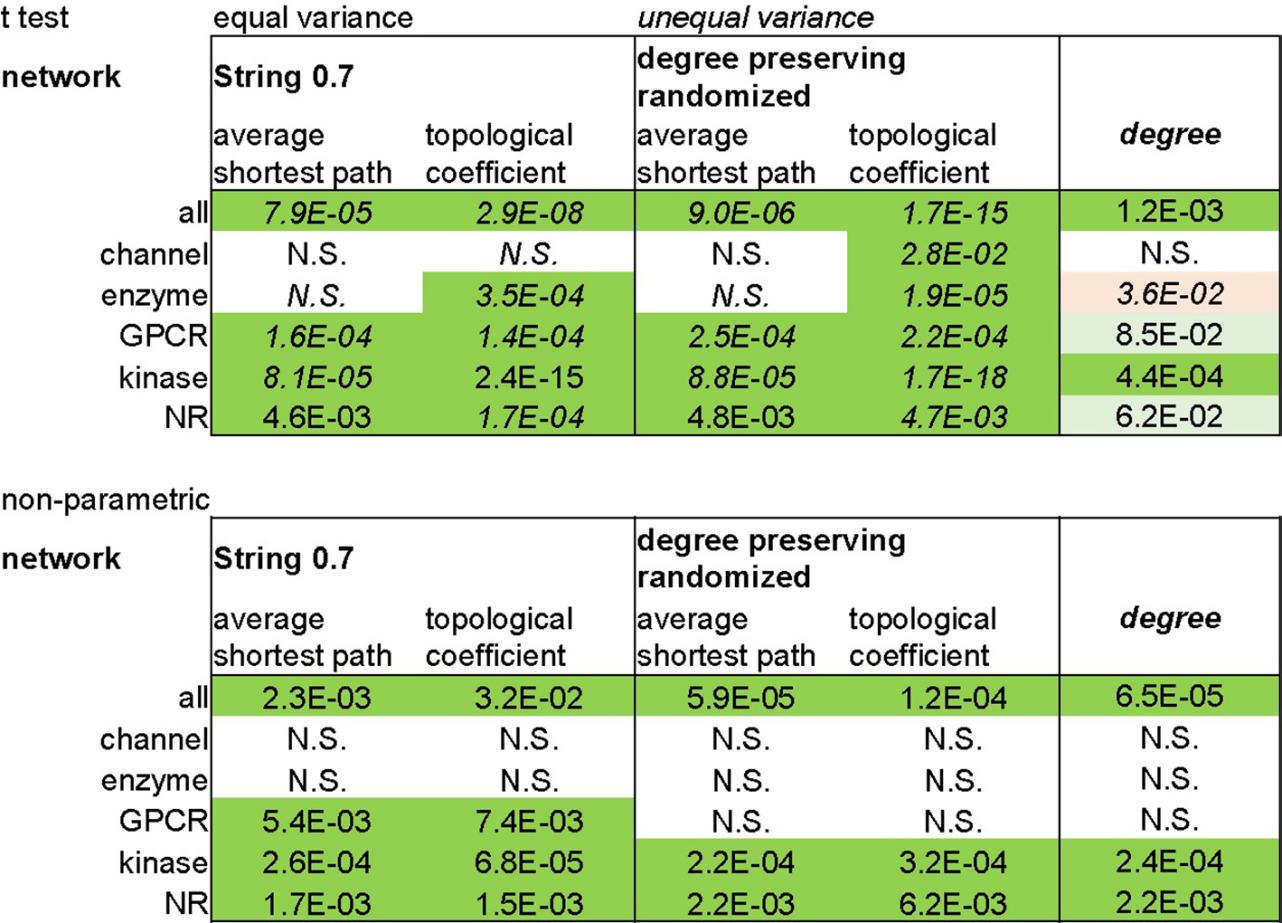
Null hypothesis probabilities (not corrected for multiple testing) of difference in centrality parameters after degree-preserving randomization across target classes

Cells are color coded according to statistical significance (α = 0.05; dark green: significant, light green: marginal significance, pink: significant—lower centrality, N.S.: not significant)

**Fig. 4:**
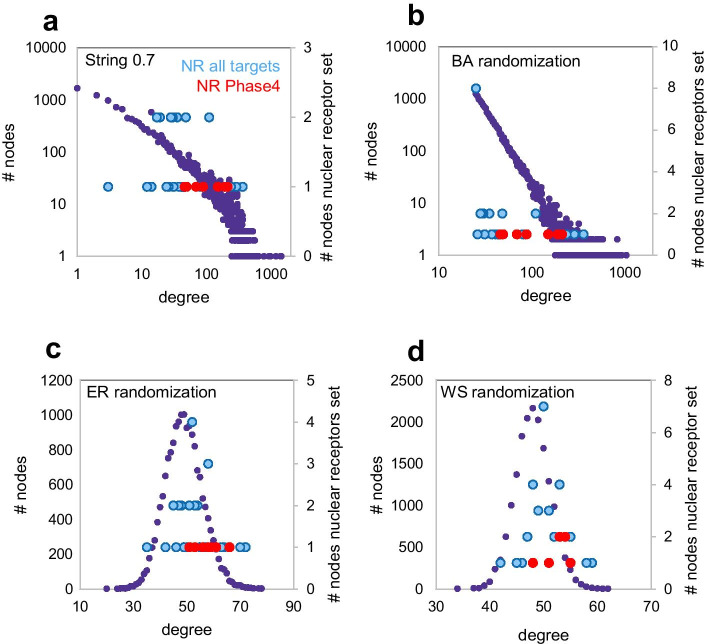
Degree distribution of Nuclear Receptors (NR) targets sets (Phase4 and all targets) plotted in the String 0.7 network (**a**) and ‘projected’ onto the degree distribution of randomized networks of comparable size (**b–d**)

To test this hypothesis we generated a Barabasi-Albert (BA) scale-free random network [45] with comparable number of nodes and edges as the String0.7 network and measured pairwise differences between random sets of nodes with degree distributions matching those of the nuclear receptors sets (Phase4 and all targets) in the original network. This target class exhibited the most consistent differences in centrality parameters across the analyzed protein networks, providing a robust control for the resilience of such differences. We ‘projected’ the original degree distributions of nuclear receptor nodes (Fig. 4a) onto the BA network (Fig. 4b, refer to Methods for details). In the BA randomized network, differences in centrality parameters observed between the two sets of nodes in the original String0.7 network retained statistical significance (Table 10). This supports the hypothesis that the differences in centrality measures between Phase4 and all targets depend on their hub position in a scale-free network structure.

**Table 10:**
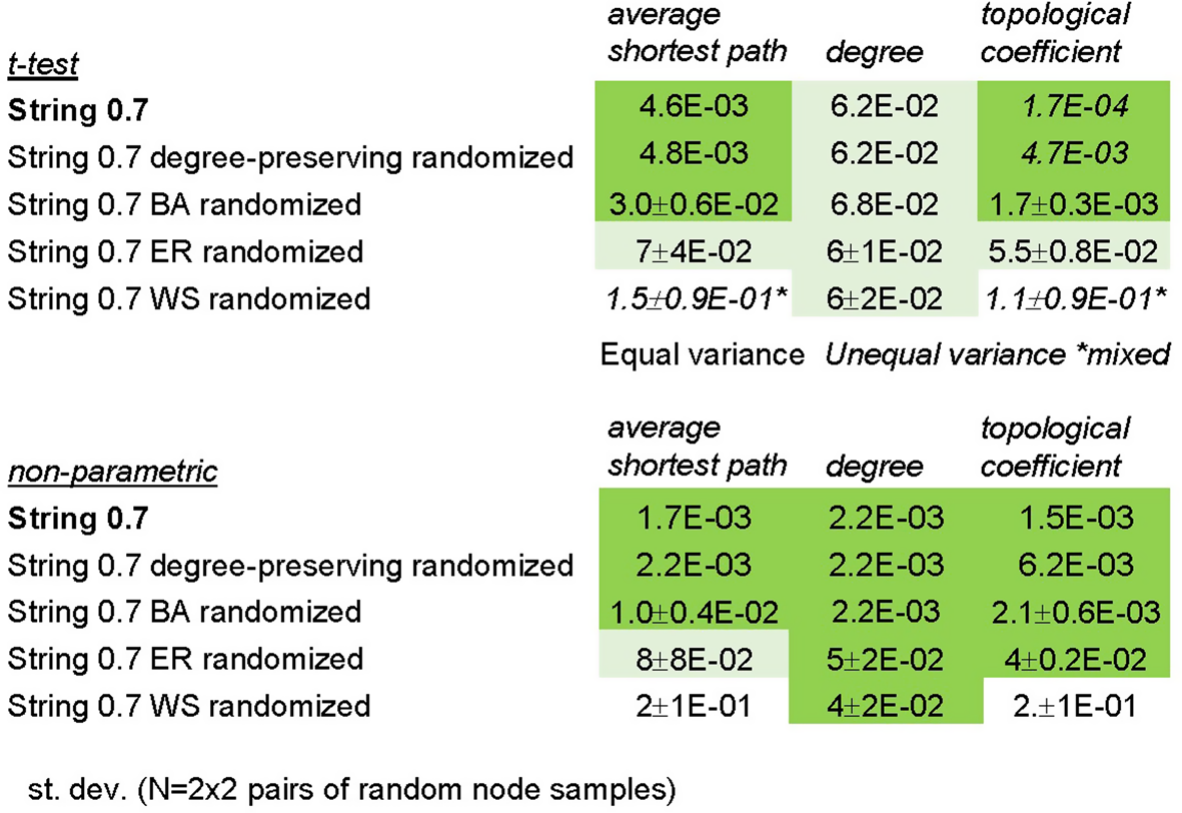
Null hypothesis probabilities (not corrected for multiple testing) of difference in centrality parameters after projection of the String0.7 degree distribution for the Nuclear Receptors targets sets (Phase4 and all targets) onto the degree distribution of randomized networks of comparable size

Cells are color coded according to statistical significance (dark green: high significance, light green: marginal significance, white: not significant

As a reverse validation of this hypothesis, we projected the degree distributions of the nuclear receptors sets (Phase4 and all targets) onto the normal degree distribution of an Erdos–Renyi (ER) random network [46] of corresponding size as the String0.7 network. ER networks lack hubs, hierarchical organization and exhibit a narrow distributions of centrality metrics (Fig. 4c, Additional file 1: Table S5, refer to Methods for details). As the degree distribution of a random network is narrower than a scale-free network, the differences in centrality parameters were reduced and lost statistical significance after ER randomization (Table 10, Fig. 4c, Additional file 1: Fig. S13), further supporting the hypothesis that the differences in centrality measures between Phase4 and all targets depend on their hub role in a scale-free network structure.

Since BA and ER networks exhibit minimal clustering (Additional file 1: Fig. S14), we performed a last randomization test by generating a Watts-Strogatz (WS) random network [47] of comparable number of nodes and edges as the String0.7 network. WS networks exhibit random features like ER networks but include a ring structure that results in higher clustering and ‘small world’ properties compared to a truly random network (Additional file 1: Fig. S14). The extent of clustering is controlled by a β parameter ranging from 0 (lattice network) to 1 (random network). We projected the degree distributions of the nuclear receptors sets (Phase4 and all targets) onto the narrow, normal degree distribution of a WS random network, generated with β = 0.25 (Fig. 4d). This network exhibited considerably higher clustering than both BA and ER randomizations (Additional file 1: Fig. S14). Once again, the differences in centrality parameters lost significance relative to the original String0.7 and BA random networks (Table 10, Additional file 1: Figs. S13, S14), supporting that a highly connected hub position, rather than clustering, determine the centrality metrics discriminating targets of selective drugs from other related proteins. To further verify that the extent of differences between centrality metrics related to different degree distributions depend on a scale-free network organization, we compared the ratios of shortest paths and topological coefficients between nodes at determined degree distribution percentiles from networks with different structures. This comparison confirmed the intuitive observation that relative differences are considerably larger in scale-free networks (truly scale-free—simulated, or approximately scale-free—real networks) compared to a random network (Additional file 1: Fig. S15).

**Fig. 5:**
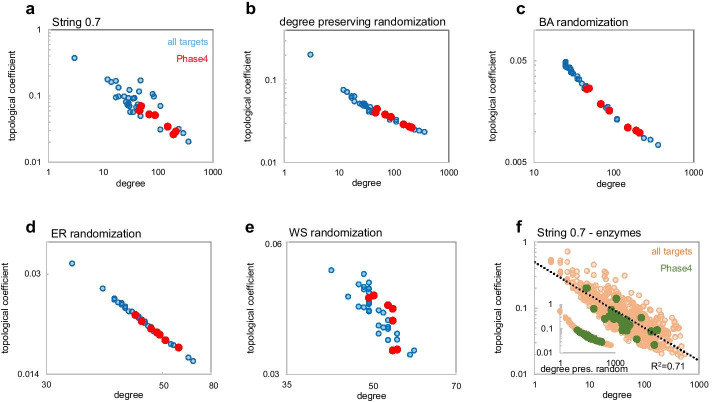
Scatter plots of topological coefficient versus degree for the nuclear receptors targets sets (Phase4 and all targets) in the String0.7 network (**a**) and projections of proportional degree distribution in randomized networks of comparable size (**b–e**). Scale free networks, as generated through degree preserving or Barabasi Albert (BA) randomization, exhibit an inflexion towards higher topological coefficients at lower degrees (**b–c**). Erdos Renyi (ER) randomization (**d**) exhibits a linear log–log correlation between the two parameters. Clustering, present in the Watts Strogatz (WS) random network (**e**), disrupts the correlation between the two parameters with a random noise effect. For the target class of enzymes (**f**), Phase4 targets exhibit significantly lower topological coefficient than all enzymes in String0.7 even if the average degree of Phase4 enzymes is lower. This effect is retained after degree preserving randomization due to the low degree inflection (inset)

To understand what determined the amplification of differences in topological coefficient relative to differences in degree between node samples in scale free networks we inspected scatter plots of these parameters for the nuclear receptor and enzyme target nodes in String0.7 and randomized networks (Fig. 5). This analysis highlighted that, in the absence of clustering, scale free networks exhibit an inflexion of increasing topological coefficient at low degree (Fig. 5b, c), resulting in amplified differences in topological coefficient relative to differences in degree by linear regression statistics. Erdos Renyi randomization (Fig. 5d) exhibits a linear log–log correlation between the two parameters, resulting in equal significance of their differences between node samples. Clustering, present in the Watts Strogatz random network (Fig. 5e), disrupts the correlation between the two parameters with a random noise effect. The target class of enzymes (Fig. 5f) is an example of synergistic effects of scale-free organization and non-random clustering amplifying the deficit in topological coefficient for Phase4 targets even if this group of protein nodes exhibited a lower average degree than all targets in String0.7 and other networks. In this case the lower topological coefficient of Phase4 nodes was retained (*t*-test) after degree preserving randomization (inset) due to the low degree inflexion of this parameter. Additionally, the effect of clustering in String0.7 results in most Phase4 nodes falling below the trendline between degree and topological coefficient, exhibiting relatively low topological coefficient at equal degree. It is plausible that clustering of enzyme nodes in String0.7 might reflect some functional network characteristics rather than mere noise as in the Watts Strogatz randomization, resulting in such biased deviation for Phase4 nodes.

### Generation of predictive models

With the analyses described so far, we produced qualitative assessments of connections relating individual node centrality parameters to a drug target’s fitness. We also addressed how entanglement between node features within annotated protein functional networks and relative abundance of literature associated to individual proteins may influence or bias these assessments. Last, we used network randomization to gain insights into the relationships between node centrality features linked to drug target proteins, identifying the number of connections (degree) of a node as their root centrality feature. This last analysis also suggested intricate relationships between centrality parameters (e.g. effect of clustering in enzymes’ degree/topological coefficient correlation, Fig. 5f). This observation indicates that underlying network characteristics discriminating drug targets from other proteins could be better defined by complex combinations of different centrality parameters rather than individual parameters. Additionally, these combinations may vary between target classes due to the different biological roles broadly associated with different protein types. In order to identify combinations of network node features that may better help identify ‘good’ drug target proteins, we generated naïve Bayesian predictive models aimed at discriminating between drug targets and other proteins based on their centrality parameters (Table 11).

**Table 11:**
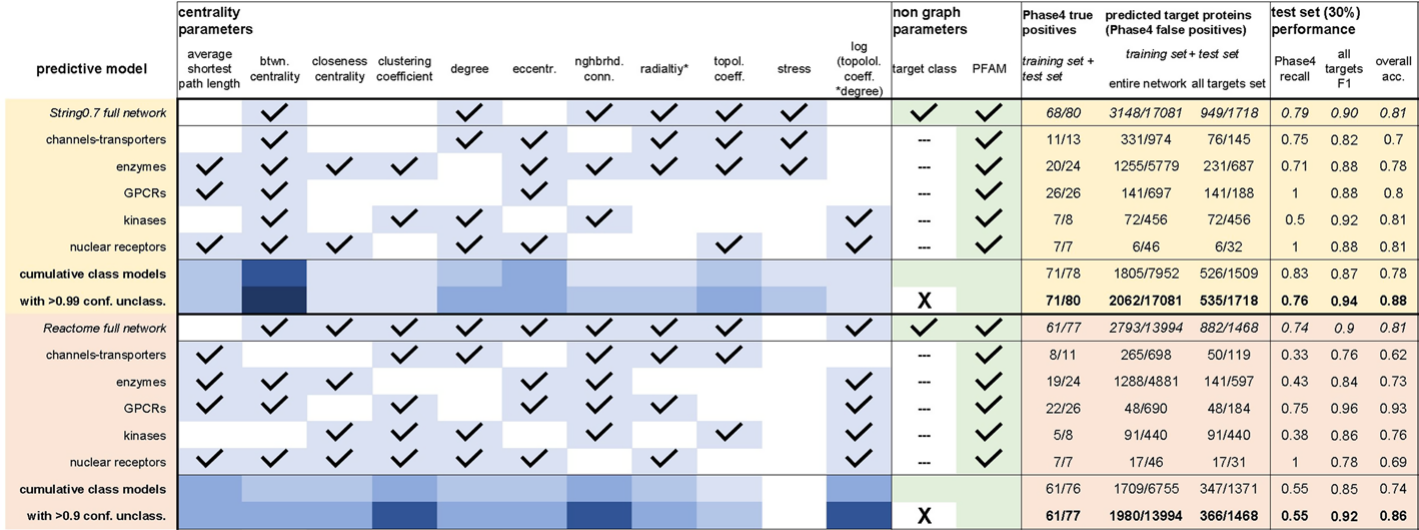
Overview of training features and performance of naïve Bayesian predictive models created from the String0.7 and Reactome networks, utilizing network-wide and target class-specific training

The latter outperformed network-wide training in both databases. Centrality features were chosen for each model with the aid of the Knime forward feature selection workflow (blue boxes on the left hand of the table). The ‘radiality’ feature (marked with an asterisk* in the table), equivalent to average shortest path or closeness centrality, performed well in several models likely due to its narrow range relative to the model training parameters

This effort was challenged by the small number of Phase4 targets relative to other proteins, which led several tested predictive model algorithms to a high type II error rate (false negatives) for Phase4 targets due to the widely greater size of one training set (non-targets) over the other (drug targets). Additionally, a perfectly accurate predictive model would be useless as it would simply discriminate between proteins that already are targets of approved drugs and ones that are not, without any predictive value for proteins that *could* become drug targets. We intended to deliberately generate models prone to a moderate extent of type I error for proteins not currently annotated as Phase4 targets. These ‘false positives’ would be predicted ‘good targets’ based on suitable combinations of their centrality parameters.

To generate such predictive models, we optimized the following performance metrics: recall (fraction of true positives that were correctly identified) for Phase4 targets, F1 measure (harmonic mean of recall and precision, precision being the fraction of true positives out of predicted positives) for other proteins, and overall accuracy (fraction of correct predictions for the total number of samples). Inclusion of Pfam [48] functional domain annotations as a non-graph feature slightly enhanced the models’ performance. Of all the analyzed networks, models generated from centrality features of String0.7 or Reactome networks performed the best (Table 11, Additional file 1: Table S6). In order to test the core hypothesis of this study that target class discrimination helps identify drug targets through deconvolution of inherent protein-class characteristics, we compared predictive models trained over entire networks versus ones trained over individual target classes. The latter performed noticeably better using distinct sets of centrality features for each target class (Table 11). As the large number of proteins that did not belong to any of the main identified target classes (classified as ‘other’) were negatively biased in these models, we empirically included a number of these proteins to our predictions by eliminating classification features from the entire network models and selecting high confidence drug target predictions for these non-class associated proteins. The final, target class specific, predictive models identified ~ 2000 proteins (~ 10% of the proteome) as potential drug targets, with good agreement between source networks from the String0.7 and Proteome networks, respectively (2043 shared predicted targets for the entire network training, contingency probability < 10^−4^; 562 shared predicted targets for individual classes training, contingency probability < 10^−4^). As an orthogonal validation, we found that these four predictive models (String0.7 and Proteome, trained either over the entire database or in a class-specific fashion), correctly identified (with a median consensus of three out of four models) 406/503 (81%) of drug target proteins annotated in a recent study [3]. The cited analysis was performed without limitations on the specificity of drugs related to individual targets (hence the considerably larger size relative to our ‘Phase4’ set of targets of highly selective drugs); the study originally reported 667 human protein targets, reduced to 503 after filtering for ‘single protein’ target annotation in ChEMBL and entry existence in the predictive model databases. The Knime [49] workflows implemented to generate these models and resulting prediction tables are available as supplementary files to this article (Additional files 2 and 3).

### Evaluation of additional non-graph descriptors

Recent analyses evaluated network characteristics of drug targets combined with annotated functional data in disease specific contexts [50, 51]. With the goal of identifying suitable drug targets in Pancreatic Ductal Adenocarcinoma, Yan et al. devised a ‘hybrid’ RNs score ranking that combined information from gene expression datasets with node centrality metrics (average shortest path length, degree) within a sub-network of the String database relevant to this disease [50]. Kim et al. identified a disease-relevant protein network (module) for Systemic Sclerosis and evaluated it with enrichment analysis of Gene Ontology (GO) biological processes descriptors [51]. This analysis identified targets which could expose vulnerabilities of the disease module for improved clinical outcome. We evaluated whether protein features external to their network representations would aid our assessment of targets’ fitness. As our analysis aims at a genome-wide evaluation of potential drug targets irrelevant of specific disease contexts, we could not rely on disease-specific annotations as done in the cited studies. We considered instead the number of disease associations for each gene, for any disease context, from curated sources in two databases: DisGeNET [52] and Genetic Association Database [53]. We then evaluated the associations of recurring GO term descriptors to Phase4 versus all target protein sets collectively and for individual target classes. Last we tested the impact of these non-graph descriptors in predictive models of targets fitness. The number of disease associations for each protein, annotated in either DisGeNET or Genetic Association Database significantly discriminated between Phase4 and all targets sets in the String0.7 and Reactome networks (the two networks utilized in our graph-based predictive models; Table 12, Additional file 1: Fig. S16a, b). We noticed however a strong association between number of disease associations and number of literature citations for these protein sets (Additional file 1: Fig. S16c, d and Table S7), possibly convoluting the differences in disease associations between Phase4 and all targets with a consequential rather than causal relationship to the fact that drug targets are extensively studied proteins, as discussed at length previously. We combined the percentile ranking of disease associations with graph centrality metrics, similarly to the cited RNs score [50] (with the difference that the original RNs score was derived from gene expression data rather than annotated disease associations) and with centrality metrics identified in this study as possible discriminants between drug targets and other proteins: topological coefficient and *log* (degree*topological coefficient). These ‘hybrid’ metrics discriminated between Phase4 and all target sets with comparable statistical significance to the number of disease associations (Table 12) yet with weaker correlation to the number of literature references (Additional file 1: Table S7), thus reducing the possible bias of consequential rather than causal associations in the evaluation of targets fitness.

**Table 12:**
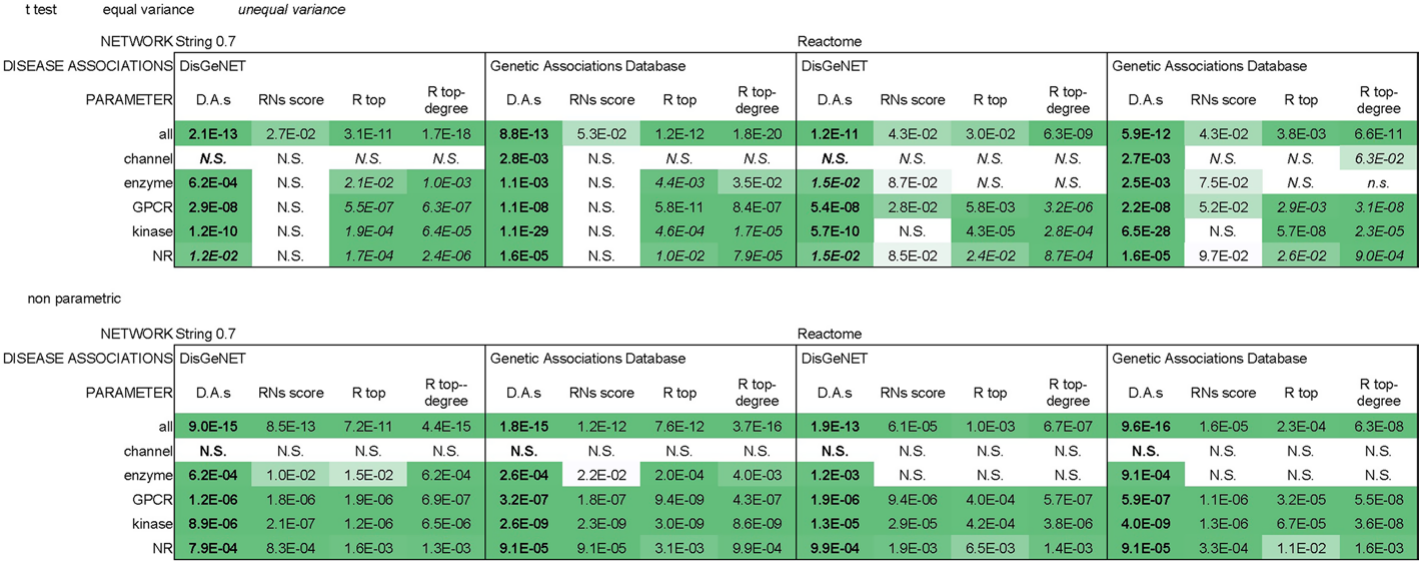
Null hypothesis probabilities for differences between Phase4 targets and all targets in disease association counts (abbreviated D.A.s) and related ‘hybrid’ graph-D.A.s parameters

Disease association counts were extrapolated from DisGeNET or Genetic Association Database. Additional parameters combining the percentile ranking of disease associations with network centrality metrics were evaluated: RNs score (reported in Ref. [50]); ‘R top’ and ‘R top-degree’ each combining the disease associations percentile (R) with one centrality parameter identified here as discriminating between ‘Phase4’ and ‘all targets’ protein sets: topological coefficient and log(degree*topological coefficient) (see Table 5), respectively. This analysis was performed on the String07 and Reactome networks, corrected for multiple testing across both centrality metric and target classes (Benjamini–Hochberg method). Cells are colored in increasingly darker shades of green according to statistical significance (N.S.: not significant)

In analyzing the GO functional descriptors associated to each protein we observed enrichment or depletion in Phase4 versus all targets sets for some of the 25 most recurring GO terms (covering 95% of all GO term associations for all nodes in the String07 network), with specific enrichment or depletion patterns for different target classes (Table 13). At the high level of a genome-wide analysis, these variations in GO terms associations may indicate underlying functional contexts that define suitable targets within various protein classes. Consideration of these associations could thus complement an evaluation of proteins’ fitness as drug targets based on their graph-centrality.

**Table 13:**
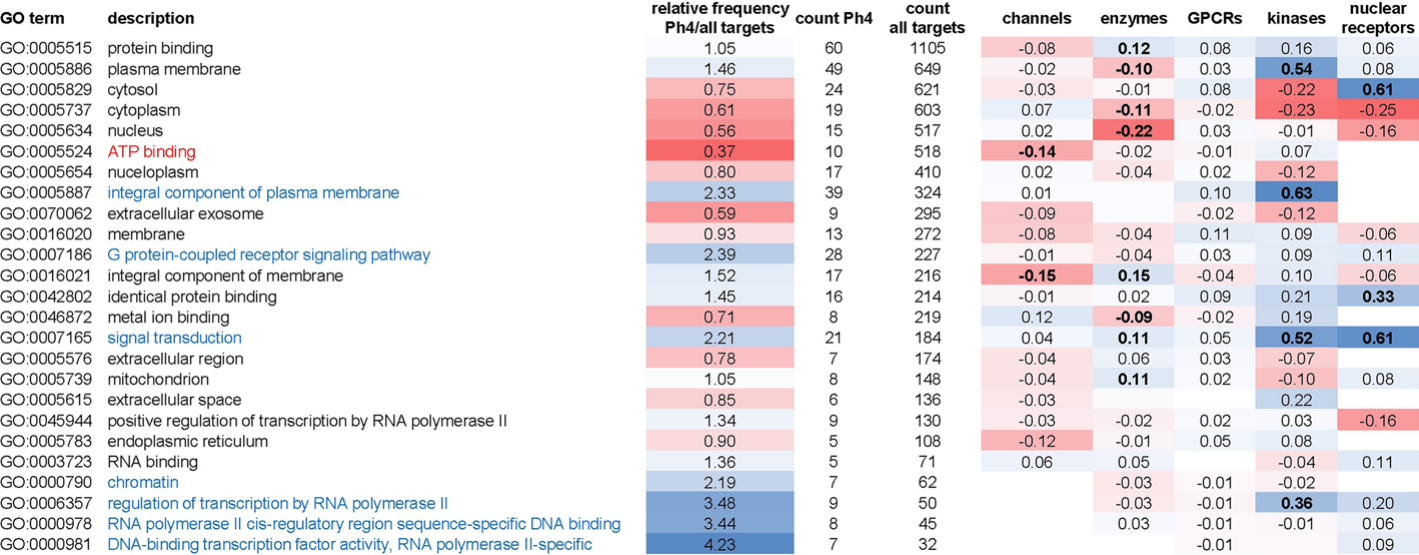
Analysis of Gene Ontology (GO) terms enrichment in ‘Phase4’ versus ‘all targets’ protein sets

The most frequent GO terms (representing 95% of all GO associations for the proteins in the String07 database) were ranked based on total number of associations. GO terms that exhibit twofold higher or lower frequency within the Phase4 set compared to the ‘all targets’ set are highlighted in blue or red respectively. In the target class specific analysis (right side of the table), the normalized difference in frequency of each term is reported. Values with effect size resulting in > 0.8 statistical power (*p* = 0.05) for the sample size of each dataset are highlighted in bold fonts

We tested the performance of our naïve Bayesian predictive models after inclusion of these additional non-graph features: number of disease associations from the DisGeNET database and GO terms (Table 14, Additional file 1: Table S6). These additional descriptors improved the performance statistics of both network-wide models based on the String0.7 and Reactome networks, and of most target class specific models without additional changes in the graph-based features selection (with the exception of the String0.7 kinase model which required selection of a different set of graph centrality features after introduction of disease association metrics). The improved statistics of predictive models that included disease associations and GO terms largely resulted from a smaller number of false positives from proteins that did not belong to the Phase4 set, thus narrowing the range of hypothetical ‘predicted’ drug targets (compare Tables 11 and 14). When benchmarked against the 503 target proteins in the dataset reported by Santos et al. [3], these more conservative predictions correctly identified 386 targets (~ 77%) with a median consensus of 2/4 models, a slightly lower recall ratio than that obtained with the models trained without disease association and GO terms features (~ 81%, median ¾ models). When combined, predictions from models that included or excluded non-graph parameters identified 428 targets (85%) with a median consensus of 5/8 models. While beyond the scope of this study, the lower number of predicted targets obtained after introduction of non-graph features brings us to speculate that their use could be fine-tuned (e.g. using selected subsets of GO terms or specific disease associations) to generate target predictions narrowly focused on specific biological and disease contexts.

**Table 14:**
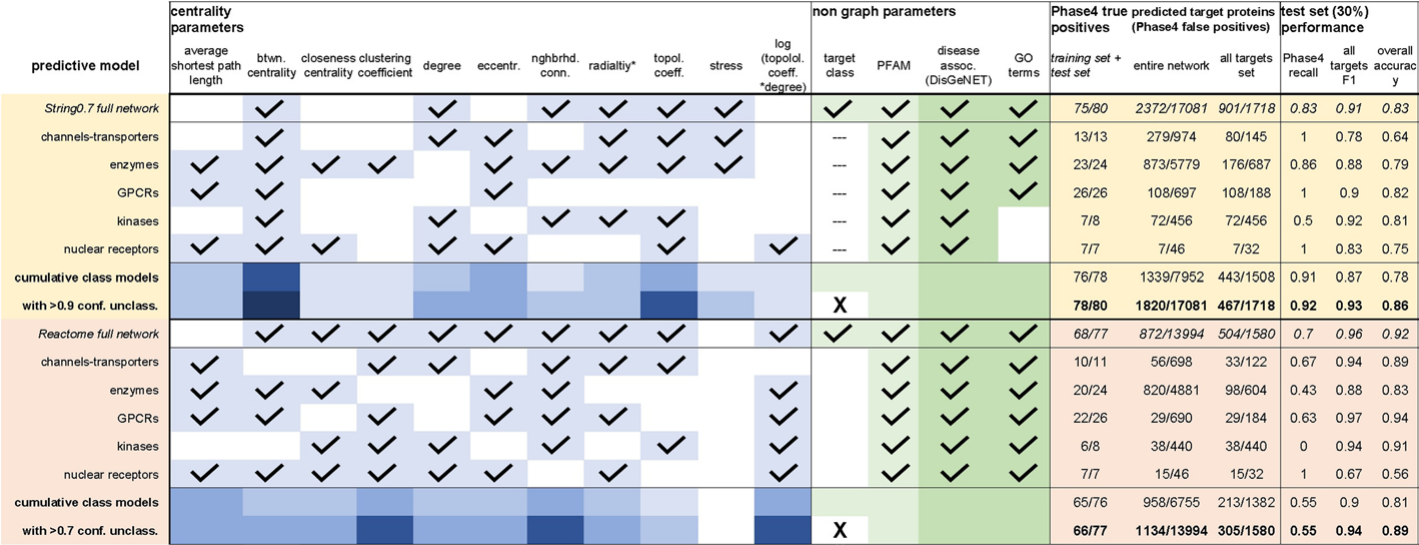
Overview of training features and performance of naïve Bayesian predictive models created from the String0.7 and Reactome networks, utilizing network-wide and target class-specific training, as in Table 11, and including additional non-graph parameters: disease association counts extracted from the DisGeNet database and GO terms associated with each protein

The inclusion of these features slightly enhanced the models’ performance (higher true positive Phase4 recall, reduced number of false positive ‘predicted’ targets)

## Discussion

In the effort to identify novel potential drug targets, cognitive bias often drives to a ‘linear’ interpretation of cellular signaling pathways neglectful of their true network-embedded nature. A bias that could drive the simplistic identification of discovery targets based on a ‘roadblock’ conception of cell signaling interference. This might lead to underestimate the risk of encountering fault resistance (lack of efficacy) or propagation (toxicity) after perturbation of targets (‘nodes’) with unsuitable positioning within their signaling network. These potential pitfalls are especially dangerous in the instance of projects based on innovative, yet poorly validated targets (e.g. from conference posters, single publications), sometimes leading to proof of concept failures even in the presence of suitable chemical matter and verified target engagement.

In our study we followed previous analyses [4–12] that identified graph node centrality as a characteristic of drug target proteins and tried to clarify some discrepancies in results from these studies. In general, the higher centrality of drug targets relative to other proteins may be a consequence of the fact that certain protein classes with inherently higher hub characteristics, or highly studied, are more likely drug targets than others. Our main finding is that this higher centrality paradigm largely holds true for targets of selective drugs versus other discovery targets within their respective protein classes. Thus, high graph centrality may be a characteristic of ‘good’ drug targets relative to functionally similar proteins, independent of any bias in graph descriptors that may derive from different protein class distributions between drug targets and other proteins. The higher node centrality of drug targets appears to be largely an inherent characteristic of their hub positioning within scale-free biological signaling network, independent of their specific local connections. However local network characteristics may also contribute to this effect, as observed for the ‘biased’ clustering of Phase4 enzymes, resulting in lower topological coefficient than nodes of comparable degree. The varying subsets of centrality features identified as predictors of drug target proteins for different protein classes further indicates that the node characteristics linked to ‘good’ drug targets in a network representation may vary between protein classes due to inherent differences in their biological functions.

Since drug targets are highly studied proteins, we evaluated the extent of knowledge bias to our observations. The outcome of this analysis is complex, indicating on one hand that networks where the higher centrality of Phase4 targets is more pronounced exhibit fewer ‘structural’ indications of bias towards highly studied proteins (i.e. lesser bias towards large nodes in degree distribution, sizeable clustering), however they exhibit higher, albeit modest, correlations between node centrality metrics and citation counts. We hypothesized that these correlations may arise in part from the implementation of text mining in the search for inferred edges included in some of the analyzed networks (i.e. String), which might introduce a bias towards recurring protein keywords. When analyzing the centrality of Phase4 targets across networks in each target class, we did not identify correlations with their relative literature enrichment. We observed however associations between the probability of higher Phase4 target centrality in specific parameters (distance, degree, topological coefficient, regardless of target classification) and the correlation between these parameters’ value and literature references within each network. A ‘combined’ parameter log (degree*topological coefficient), exhibiting differences between Phase4 and all targets in several target classes and networks, abrogated such association with the relative literature enrichment of target nodes. Our interpretation of these analyses is that the identified associations between literature enrichment and node centrality fail to demonstrate a direct causal relationship determining network centrality for certain proteins simply because they are highly studied. These correlations may be conversely interpreted as coincidental consequences of the fact that highly studied proteins could indeed *be* hubs in biological networks. However, as novel therapeutic concepts may involve target proteins that have not been studied as extensively as targets of approved drugs, a careful evaluation of their specific network connections, source and literature references would be pertinent, in order to assess the relevance of their node parameters as indicators of their potential fitness as drug targets. Additionally, as some studies identified prominent node centrality in network representations with essential proteins and possibility of toxic effects upon their targeting with pharmaceutics [22, 24], we would recommend an especially careful evaluation of toxicity liabilities for hypothetical targets which exhibit marked hub features.

To complete our analysis, we generated predictive models of ‘likely’ drug targets utilizing the node centrality features from two of the studied protein networks (String0.7 and Proteome). These models, recognizing over 80% of ‘true’ selective drugs targets (Phase4 set), also identify a limited number of ‘false positives’ (~ 10% of the proteome, defined as non-redundant human coding genome in Uniprot; Table 3). Which we interpret as likely ‘fit’ targets based on their centrality metrics. As our ‘true’ target training set was deliberately limited to protein targets of very selective drugs, these predictions encouragingly largely overlap with known targets of drugs with less restrictive selectivity profiles. The inclusion of non-graph features such as disease associations or functional annotations may further enable predictive models fine tuned on specific biological or disease contexts.

## Conclusions

‘Druggability’ evaluations of potential targets from a structural biology perspective [54] are routinely performed in the pharmaceutical industry. These evaluations assess the availability of structural information, presence of surface cavities or pockets suitable for ligand binding and their physico-chemical properties. There are several other criteria that inform the evaluation of a drug target candidate, often target and business specific. In some cases, the perceived accessibility of a ‘druggable’ target may overcome incomplete validation of its therapeutic rationale, based on the reasonable assumption that the swift development of ‘tool compounds’ could help bridge this gap during early discovery stages. Nevertheless, a dispersion of resources will occur whenever the therapeutic hypothesis is disproved once suitable tool compounds are obtained. In the absence of a compelling proof of concept for the underlying therapeutic hypothesis, the assessment of a protein’s network centrality could provide an easily available additional piece of information that might aid in the decision of whether to pursue this protein as a discovery target or not, and consequent allocation of resources.

## Methods

### Datasets retrieval and assembly

The ‘Phase4 targets’ and ‘all targets’ protein sets were identified within the ChEMBL database [32] (version 27, 2020; https://www.ebi.ac.uk/chembl/). ‘Phase4 targets’ were identified as follows: the ‘*compounds*’ database was filtered for ‘*type* = *small molecules*’, ‘*max phase* = 4’ ‘*targets* ≤ 4’. The ‘*browse drug mechanisms*’ analysis was performed for the resulting set of 538 compounds including the following filters: ‘*target organism* = *homo sapiens*’ and ‘*target type* = *single protein*’, leaving 80 unique proteins. ‘All targets’ were identified as follows: the ‘*targets*’ database was filtered as above for human, single protein targets. Additionally, to ensure analysis of bona fide investigational – discovery targets, only targets with ≥ 40 associated compounds were selected, leaving 1743 proteins (excluding overlapping Phase4 targets). ChEMBL target identifiers were translated to their matching Uniprot [44] identifiers using the ‘ChEMBL_uniprot_mapping.txt’ file downloaded from the ChEMBL web interface. Searches of the StringDB [34] (v11.0, 2020; https://String-db.org/) with the ChEMBL and Uniprot identifiers further retrieved the corresponding String identifiers. The functional classification of targets based on Gene Ontology [33] (GO) terms was performed using the StringDB GO-terms analysis tool applied to ‘molecular function’ annotations, pooling proteins by the following logical combinations of identifiers: channel *or* transporter (class ‘channels and transporters); enzyme *not* kinase (class ‘enzymes’); GPCR *or* G-protein coupled receptor (class ‘GPCRs’); kinase (class ‘kinases’); nuclear receptor (class ‘nuclear receptors’). The complete set of GO annotations for the Uniprot reference human proteome (goa file) was downloaded from the Gene Ontology Consortium web page (http://current.geneontology.org/products/pages/downloads.html) The number of citations for each target protein was retrieved from automated PubMed searches (https://pubmed.ncbi.nlm.nih.gov) using the proteins gene abbreviation as search term applying the filters: ‘human’, ‘journal article’ and ‘title-abstract’. Search automation was implemented using a Knime [49] ‘GET Request’ node workflow. Disease association annotations were downloaded from the DisGeNet web page (curated gene-disease associations) (https://www.disgenet.org/downloads#) and Genetic Association Database web page (https://geneticassociationdb.nih.gov/).

### Functional protein interaction databases retrieval and network analysis

Network analysis was performed using Cytoscape v3.8 [55]. Networks were either downloaded separately and imported (String v11.0 [34], InBioMap v1 [43]) or directly imported using the NDEx web import function in Cytoscape (BioGRID v3.5 [28], HumanNet XN v2 [41], Reactome v71 [42]). The complete human (taxonomy ID = 9606) String network was downloaded from the StringDB website and filtered for different edge confidence cutoffs using the ‘zgrep’ editing command (e.g., for generation of the network at 0.7 confidence cutoff: *zgrep* “^” *9606.protein.links.v11.0.txt.gz | awk* ‘($3 > 700)’ > *highconf_links.txt*). String networks contain a duplicate number of undirected edges as each connection is listed twice in the network file (i.e. A–B and B–A), giving rise to a degree distribution in multiples of 2. While this does not affect the direct network analysis, it has consequences for network randomization as it implies reassignment of a duplicate number of edges. In the analysis descriptions and randomization tests within this study we considered the String network with single undirected edges between each node pair (i.e. A–B only). Analysis of node parameters was performed using the Cytoscape network analysis tool [35]. Nodes with topological coefficient = 0, were excluded from statistical analysis of this parameter, as this corresponds to nodes with a single edge (degree = 1), confounding the trend of decreasing topological coefficients at increasing node degree. The percentages of such excluded nodes in the various analyzed full networks were as follows: String0.7 = 9.8%; String0.9 = 14.8%; String0.5 = 1.1%; BioGRID = 16.8%; HumanNet XN = 2.6%; Reactome = 11.5%; InBioMap = 6.6%.

### Full networks parameters distribution analysis

To evaluate overall characteristics and differences between the analyzed functional protein interactions networks, we mainly focused on the analysis of node degree distribution. A simple evaluation of scale-free characteristics was performed by power law fitting of node count vs degree for a degree range k ≥ 100, ≤ 1000 (to eliminate bias in the fitting slope caused by low degree saturation). The above fitting procedure results in an imbalanced statistical weight of low degree versus high degree nodes. A more advanced fitting procedure was performed utilizing cumulative degree probabilities to avoid the uneven statistical weight at different degree ranges in simple scatter plots. The fitting equation (*Eq. 4.48 in Ref* [17]*.*) accounts for low degree saturation and high degree cutoff through limit ‘k_sat_’ and ‘k_cut_’ parameters, respectively. All networks fit well to this model with exponential restrained in the scale-free range, between 2 and 3. No high degree k_cut_ correction was applied. The low degree k_sat_ cutoff was considerably larger for the String0.5 network and HumanNet XN than other networks, confirming the more pronounced low-degree saturation hypothesized from visual inspection of scatter plot distributions.

### Statistical analysis and data visualization

Statistical analysis and data visualization were performed using Microsoft Excel®, including the ‘Solver’[56] and ‘Real Statistics’[57] add-in packages (f test, t test, non-parametric test, least squares fitting, linear regressionα and ANOVA, Benjamini–Hochberg multiple sampling correction, 2D plots) or TIBCO Spotfire® (ANOVA, Spearman rank order R^2^, contingency tables χ^2^, box and whisker plots, 3D plots). The following statistical evaluations were performed according to the listed scenarios. ANOVA was used for comparison of node descriptors ranges between multiple target classes and evaluation of linear regression between literature citations and differences in centrality parameters for various target classes. T test (one tailed), equivariant or unequal variance depending on differences in sample size and variance (verified with F test), and Mann Whitney non-parametric exact test [58] (to account for the asymmetric, non-normal distribution of analyzed parameters) were used for pairwise comparisons between Phase4 target lists vs all targets lists (class sorted). Mann Whitney non-parametric test with randomized simulated data [59] (N = 1000) was used to evaluate the *P* value of low-degree saturation power law fits to the cumulative degree distribution probabilities of the analyzed networks. Benjamini–Hochberg correction was applied by adjusting the raw probabilities to the ratio between correction factor and threshold α (0.05). Spearman rank order R^2^ was used to evaluate correlations between citation counts and node centrality parameters. Contingency table χ^2^ were calculated to evaluate categorical enrichments (i.e. in predictive models).

### Network randomization

Network randomization was performed using the Cytoscape Network Randomizer application [60] (v 1.1.3). Random networks were generated based on the size of the String network at 0.7 confidence cutoff (17,161 nodes, 419,761 edges, average degree—K_mean_ 48.92). Barabasi-Albert randomization was performed with N = 17,161 and m = m0 = 25, yielding a total of 427,825 edges (a ratio of 1.02 relative to the original String0.7 network), K_mean_ = 49.86), Erdos–Renyi randomization (n, M model) was performed with N = 17,161 and M = 419,761 (consequently yielding equal K_mean_ to the original String 0.7 network). Watts–Strogatz randomization was performed with N = 17,161, K_mean_ = 49 and β = 0.25, resulting in a network with 411,864 total edges (a ratio of 0.98 relative to the original String0.7 network).

### Projection of degree distributions from scale-free to random networks

Projection of the original network degree distributions of the ‘nuclear receptors—Phase4’ and ‘nuclear receptors—all targets’ protein sets onto the scale free BA network, which had a higher minimum degree (K_min_) than the String0.7 network, all the nodes with K(String) < K_min_ (BA) were assigned value = K_min_ (BA); additionally, due to the incomplete frequency of individual high degree values, missing K(String) values were transferred to the nearest value in the BA randomized network.

Projections of the original network degree distributions of the ‘nuclear receptors—Phase4’ and ‘nuclear receptors—all targets’ protein sets onto the normal degree distribution of random networks were obtained by selecting in each random network two pairs of random sets of nodes of equal size as the nuclear receptors sets (Phase4, N = 7; all targets, N = 32). Each random set met the following criteria (see Additional file 1: Table S5 for specific parameter values of each random sample):

$${\text{K}}_{{{\text{mean}}}} random \approx {\text{K}}_{\min } random + \left( {{\text{K}}_{\max } random{-}{\text{K}}_{\min } random} \right) \cdot \log \left( {{\text{K}}_{{{\text{mean}}}} String0.7} \right)/\log \left( {{\text{K}}_{\max } String0.7} \right);$$

standard deviation K_N_*random* ≈ (K_max_*random* – K_min_*random*) • standard deviation [log(K_N_*String0.7*)].

This procedure approximates a log scaling of the original String0.7 degree distribution but circumvents the variation in relative distributions after scaling caused by mean(logK_N_) < log(K_mean_). Normality of the selected random sets was verified using the Shapiro–Wilk test.

### Generation of predictive models

We designed Knime [49] workflows to evaluate network centrality features and integrate them in predictive models aimed at identifying likely drug targets from the ‘Phase4’ versus ‘all targets’ classification used through this study. We tested all the predictive models from the Knime ‘Analytics’ node repository (i.e. Probabilistic Neural Network, Decision Tree, Random Forest, Naïve Bayesian model). We observed that most tested algorithms, with the exception of the Naïve Bayesian method, were heavily biased by the larger size of the ‘all targets’ proteins set compared to the ‘Phase4’ set, resulting in nearly 100% type II error (false negatives) for Phase4 targets, regardless of adjustment of the prediction settings. We thus opted for the optimization of Naïve Bayesian models with the aid of ‘forward feature selection’ and ‘backwards feature elimination’ meta-nodes to identify suitable centrality features. Datasets were randomly split in 70% training – 30% test sets using the ‘Partitioning’ node, ensuring proportionality between ‘Phase4’ and ‘all target’ sets. We tested predictive models for all the protein networks analyzed in this study, finding that only models derived from the String0.7 and Reactome networks performed satisfactorily according to our optimization metrics focused on Phase4 recall, all targets F1 parameter (harmonic mean of recall and precision) and overall accuracy. Models were trained over the entire networks or individual target classes, as summarized in Tables 11 and 14. The Knime workflows implemented to generate these models and resulting prediction tables are available as supplementary files to this article (Additional files 2 and 3).

### GO term enrichment analysis

The full list of GO annotations for the human proteome (goa file) was uploaded in a Knime workflow where GO terms were ranked by frequency and filtered to include 95% of all associations, resulting in 25 high frequency GO terms. Gene identifiers were matched to the String07 network and GO terms associations were enumerated by target status (phase4 and all targets sets) and protein class. Relative enrichment or depletion of terms between protein groups was assessed by evaluating the normalized ratios and differences between individual GO terms associated to different groups of proteins (e.g. ‘protein binding’ for Phase4 enzymes vs. all targets enzymes). A statistical analysis of these differences was performed by assessing the statistical power of the observed effect sizes relative to the sample size of each group of proteins. Differences exhibiting > 0.8 statistical power with target *P* = 0.05 were noted as meaningful and highlighted in Table 13.

## Supplementary Information

**Additional file 1.** Supplementary Figures 1 to 15, Supplementary tables 1 to 7.

**Additional file 2.** Excel spreadsheet containing network analysis data, GO term definitions and output tables for predictive models of drug target proteins.

**Additional file 3.** Knime workflows utilized to generate predictive models of drug target proteins from network centrality metrics, GO terms and annontated disease associations.

## Data Availability

The datasets analysed during the current study are available in the following repositories: ChEMBL (https://www.ebi.ac.uk/chembl/); Uniprot (https://www.uniprot.org/); StringDB (https://String-db.org/); PubMed (https://pubmed.ncbi.nlm.nih.gov); InBioMap (https://inbio-discover.com/); BioGRID (https://thebiogrid.org/); HumanNet (https://www.inetbio.org/humannet/); Reactome (https://reactome.org/); DisGeNET (https://www.disgenet.org/); Genetic Association Database (https://geneticassociationdb.nih.gov/). All data generated or analysed during this study are included in this published article [and its additional files].
