# Supplementary material for: Centrality of drug targets in protein networks
Source: BMC Bioinformatics. 2021 Oct 29;22:527. doi: 10.1186/s12859-021-04342-x (PMC8555226; doi:10.1186/s12859-021-04342-x)
Supplement: Supplementary file 1 — Additional file 1. Supplementary Figures 1 to 15, Supplementary tables 1 to 7. [file 12859_2021_4342_MOESM1_ESM.pdf]

SUPPLEMENTARY FIGURES AND TABLES

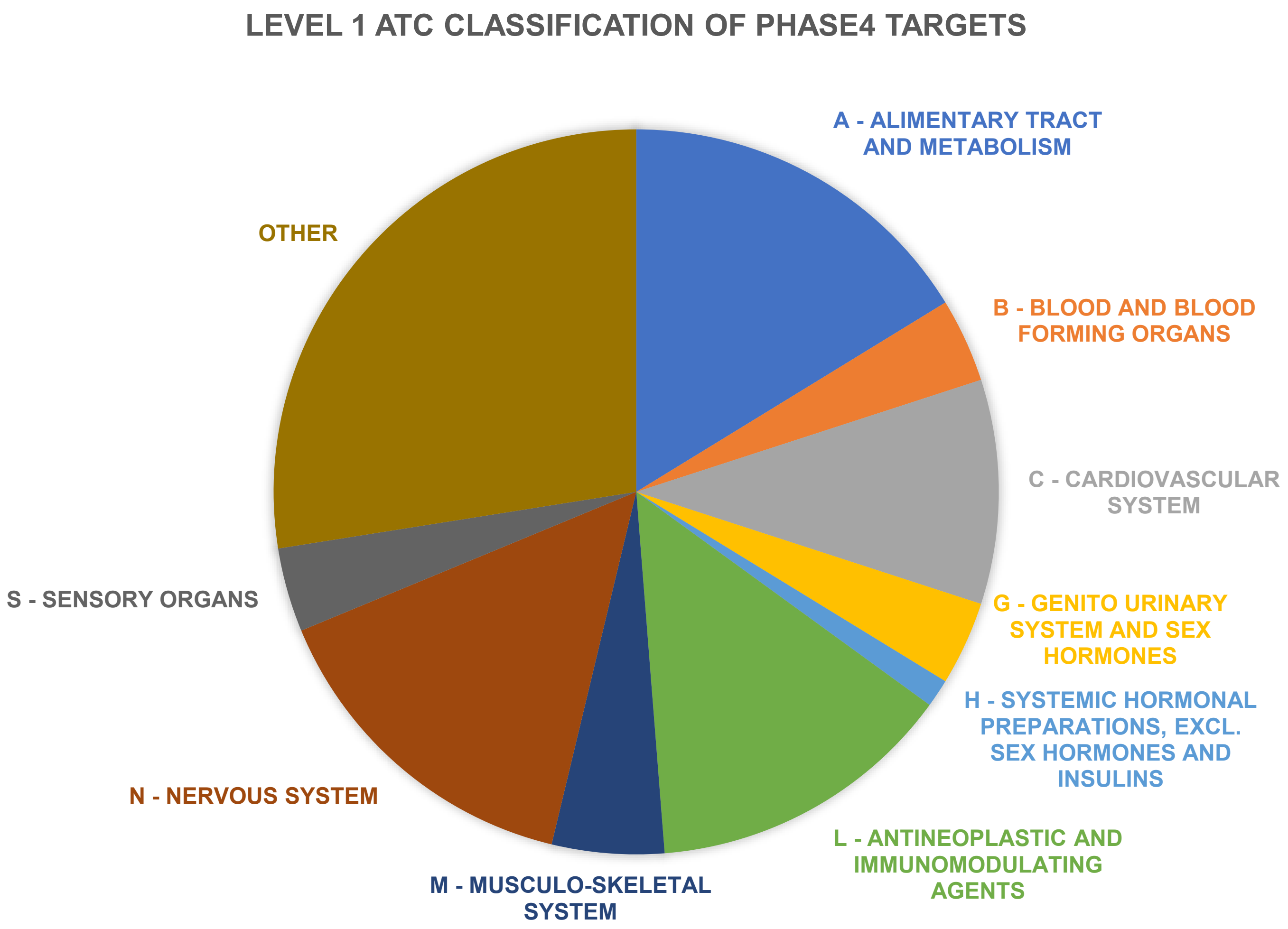

Figure S1. Anatomical Therapeutic Chemical (ATC) classification of 'Phase4' targets

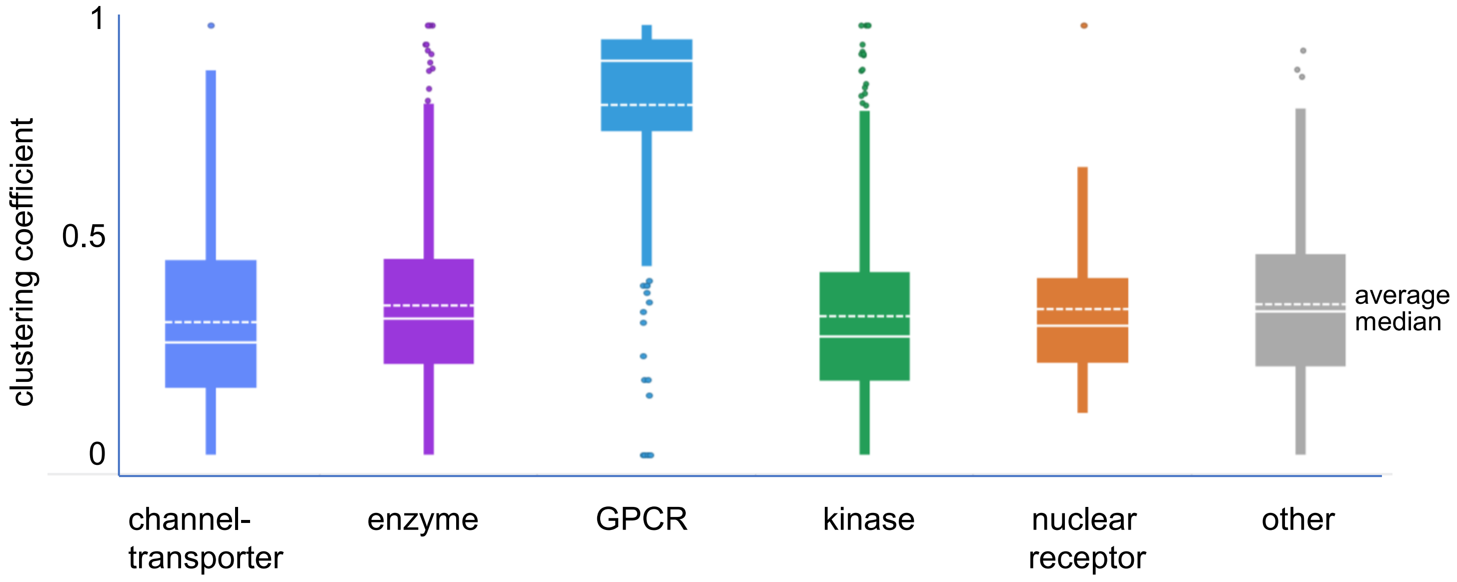

Figure S2. Different clustering coefficient distribution between target classes in the String0.7 network.

**Centrality Metric**	**p-value**
clustering coefficient	1.8E-167
neighborhood connectivity	5.0E-141
degree	1.4E-74
average shortest path	9.5E-23
closeness centrality	1.1E-21
eccentricity	1.8E-9
stress	8.8E-6
topological coefficient	3.9E-4
betweenness centrality	6.7E-1

Size dependent parameters

Normalized parameters

Table S1. ANOVA analysis of centrality parameters distribution across target classes in the String0.7 network, listed in decreasing order of variation.

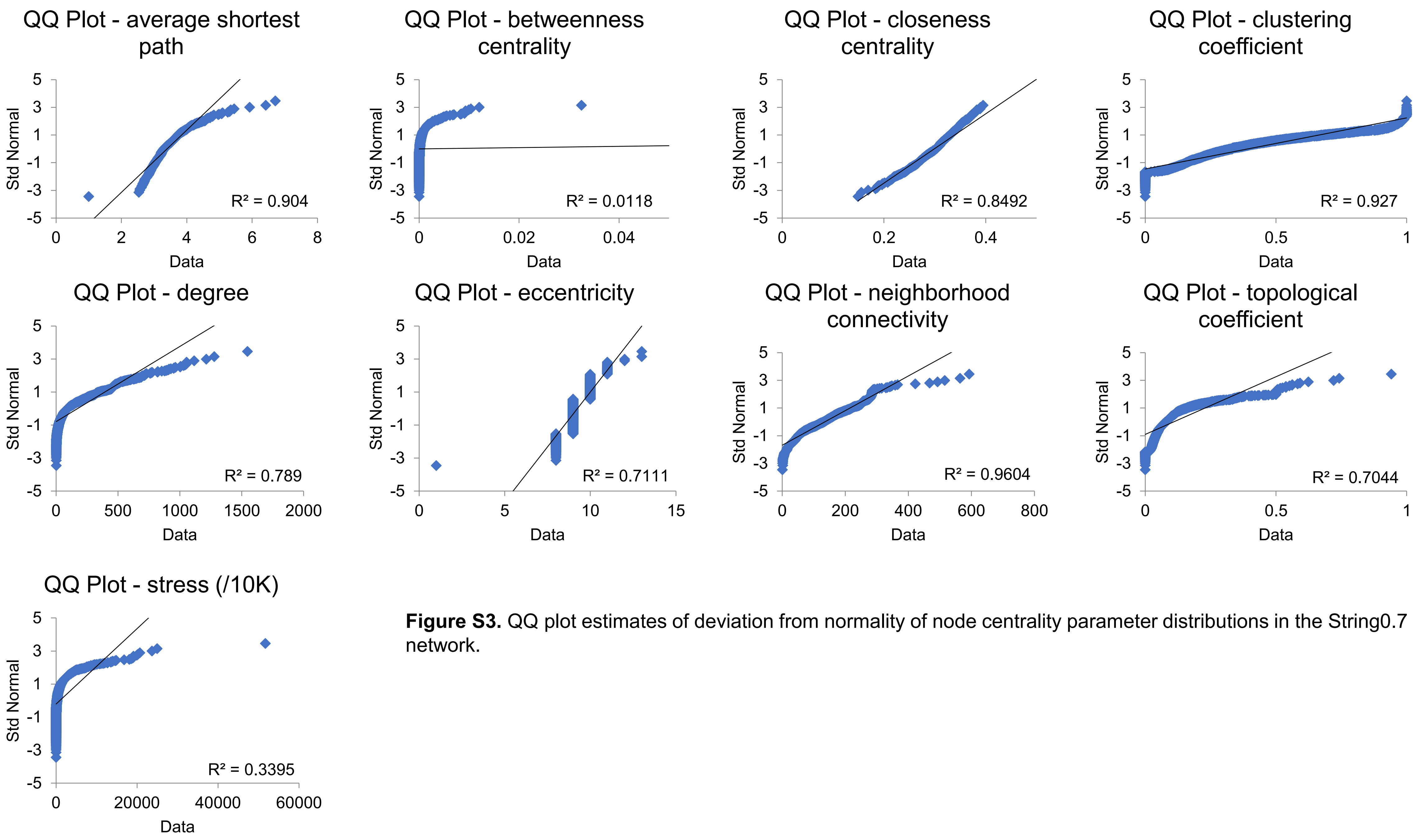

Figure S3. QQ plot estimates of deviation from normality of node centrality parameter distributions in the String0.7 network.

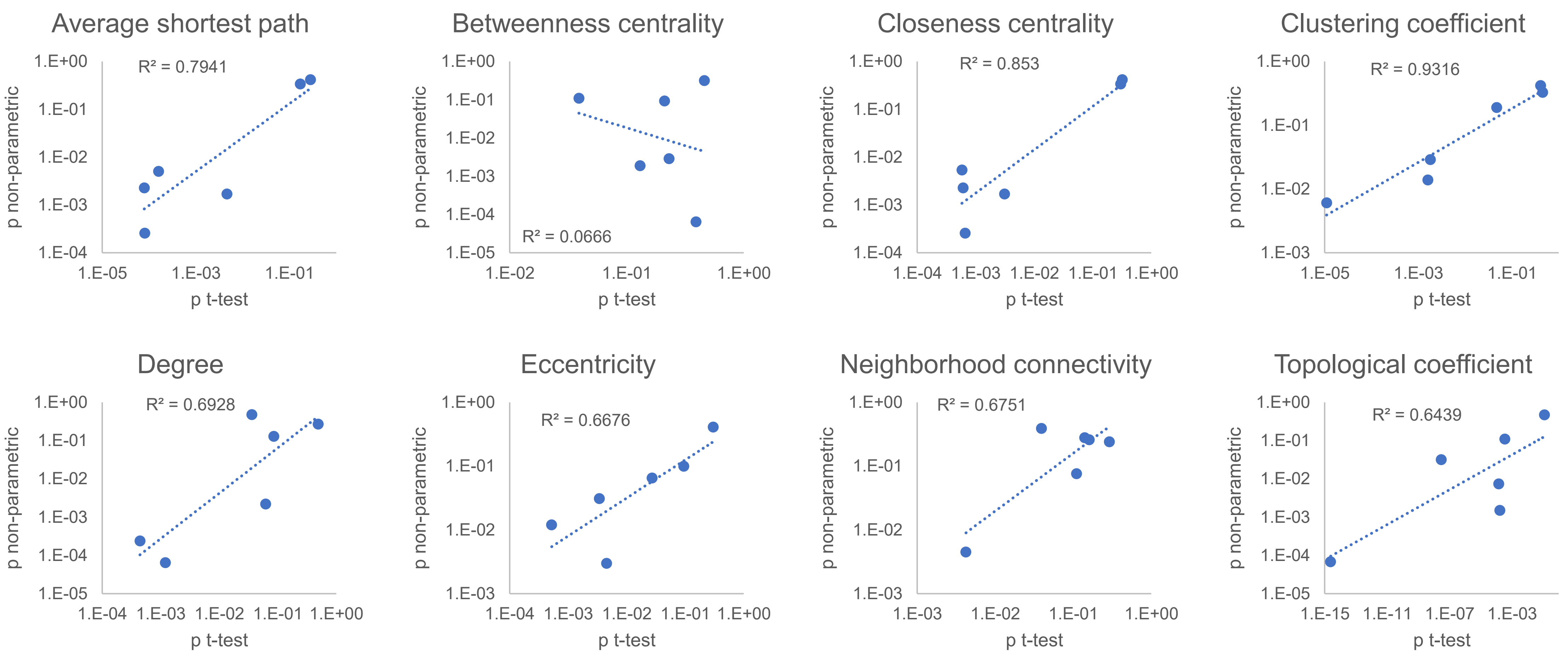

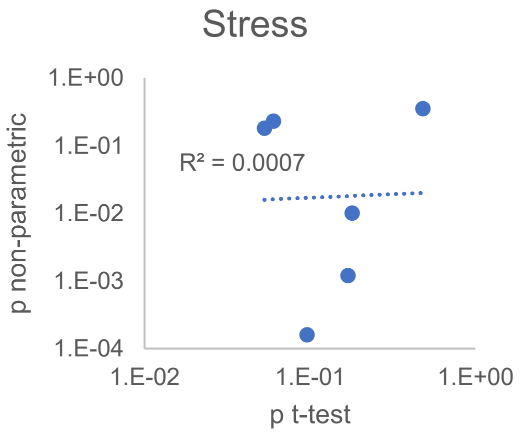

Figure S4. Pairwise correlations between linear regression and non-parametric raw probabilities (not corrected for multiple testing) in centrality parameters difference between Phase4 targets and all targets in the String0.7 network. Correlations break down completely only for parameters that exhibited extreme deviations from normality (Betweenness centrality and Stress, Figure S3)

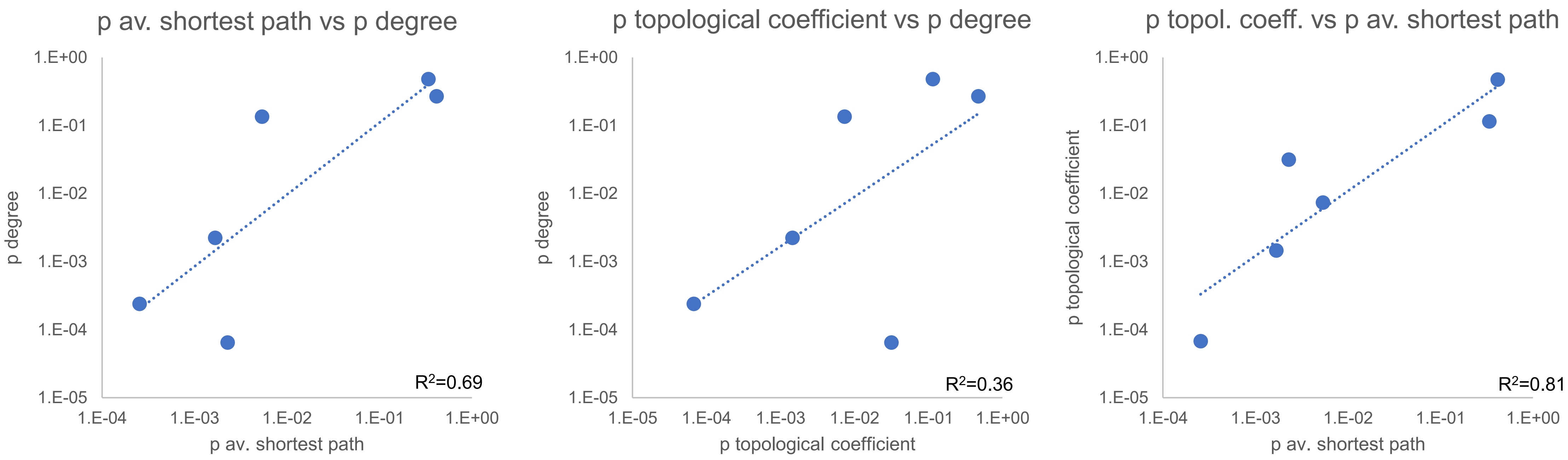

Figure S5. Pairwise correlations between probabilities (non-parametric, not corrected for multiple testing) in centrality parameters difference between Phase4 targets and all targets in the String0.7 network.

network	String 0.7		String 0.9		String 0.5		BioGRID		HumanNet XN		Reactome		InBio_Map	
	phase 4	all targets	phase 4	all targets	phase 4	all targets	phase 4	all targets	phase 4	all targets	phase 4	all targets	phase 4	all targets
all	80	1718	77	1565	80	1743	78	1681	80	1715	77	1580	80	1719
channel	13	145	10	115	13	145	13	135	13	143	11	122	13	139
enzyme	24	688	24	632	24	694	24	678	24	681	24	604	24	686
GPCR	26	188	26	179	26	189	24	158	26	188	26	184	26	182
kinase	8	456	8	413	8	471	8	468	8	459	8	440	8	469
nuclear receptor	7	32	7	32	7	32	7	32	7	32	7	32	7	32

Table S2. Target class composition of analyzed networks

overlap	String 0.7	String 0.9	String 0.5	BioGRID	HumanNet XN	Reactome	InBioMap
String 0.7		1.00	0.99	0.99	0.99	0.99	0.97
String 0.9	0.91		0.90	0.91	0.90	0.93	0.90
String 0.5	1.00	1.00		1.00	1.00	1.00	1.00
BioGRID	0.96	0.97	0.96		0.97	0.97	0.97
HumanNet XN	0.98	0.98	0.98	0.99		1.00	0.98
Reactome	0.91	0.94	0.91	0.92	0.92		0.91
InBioMap	0.97	0.99	0.99	0.99	0.99	0.99	

Table S3. Relative overlap between analyzed networks, limited to the drug targets datasets (Phase4 + all targets)

**degree**	String 0.7	String 0.9	String 0.5	BioGRID	HumanNet XN	Reactome	InBioMap
String 0.7		0.95	0.9	0.1	0.07	0.38	0.47
String 0.9			0.74	0.09	0.04	0.35	0.44
String 0.5				0.14	0.1	0.4	0.47
BioGRID					0.39	0.26	0.38
HumanNet XN						0.23	0.23
Reactome							0.41
InBioMap							

**shortest path**	String 0.7	String 0.9	String 0.5	BioGRID	HumanNet XN	Reactome	InBioMap
String 0.7		0.75	0.86	0.18	0.21	0.45	0.26
String 0.9			0.56	0.15	0.17	0.44	0.19
String 0.5				0.22	0.26	0.46	0.31
BioGRID					0.56	0.28	0.62
HumanNet XN						0.35	0.55
Reactome							0.32
InBioMap							

**topological coefficient**	String 0.7	String 0.9	String 0.5	BioGRID	HumanNet XN	Reactome	InBioMap
String 0.7		0.4	0.61	0.13	0.22	0.22	0.19
String 0.9			0.24	0.11	0.14	0.2	0.16
String 0.5				0.12	0.27	0.16	0.14
BioGRID					0.28	0.15	0.28
HumanNet XN						0.21	0.22
Reactome							0.21
InBioMap							

**clustering coefficient**	String 0.7	String 0.9	String 0.5	BioGRID	HumanNet XN	Reactome	InBioMap
String 0.7		0.47	0.61	0.01	0.04	0.25	0.16
String 0.9			0.31	0.04	0.06	0.2	0.13
String 0.5				0	0.05	0.21	0.17
BioGRID					0	0	0.02
HumanNet XN						0.04	0.06
Reactome							0.15
InBioMap							

**between centrality**	String 0.7	String 0.9	String 0.5	BioGRID	HumanNet XN	Reactome	InBioMap
String 0.7		0.59	0.71	0.2	0.22	0.31	0.25
String 0.9			0.49	0.19	0.22	0.32	0.26
String 0.5				0.23	0.24	0.32	0.3
BioGRID					0.38	0.32	0.47
HumanNet XN						0.29	0.36
Reactome							0.42
InBioMap							

**neighborhood connectivity**	String 0.7	String 0.9	String 0.5	BioGRID	HumanNet XN	Reactome	InBioMap
String 0.7		0.78	0.76	0.01	0	0.18	0.08
String 0.9			0.59	0	0.01	0.15	0.07
String 0.5				0.01	0.02	0.19	0.07
BioGRID					0.05	0	0.2
HumanNet XN						0	0.05
Reactome							0
InBioMap							

**stress**	String 0.7	String 0.9	String 0.5	BioGRID	HumanNet XN	Reactome	InBioMap
String 0.7		0.61	0.74	0.2	0.22	0.37	0.3
String 0.9			0.53	0.18	0.19	0.33	0.29
String 0.5				0.23	0.24	0.39	0.36
BioGRID					0.38	0.27	0.47
HumanNet XN						0.27	0.38
Reactome							0.4
InBioMap							

AVERAGE	String 0.7	String 0.9	String 0.5	BioGRID	HumanNet XN	Reactome	InBioMap
String 0.7		0.65	0.74	0.12	0.14	0.31	0.24
String 0.9			0.49	0.11	0.12	0.28	0.22
String 0.5				0.14	0.17	0.30	0.26
BioGRID					0.29	0.18	0.35
HumanNet XN						0.20	0.26
Reactome							0.27
InBioMap							

Table S4. Pairwise Spearman rank order correlations of different node parameters between the different analyzed networks. Boxes are color coded in a blue-white-red scale ranging from high to low correlation. Correlations vary across parameters and are intuitively higher between the three String networks mapped at difference edge confidence levels. Marked differences between the various networks likely reflect the different annotation procedure implemented in their assembly.

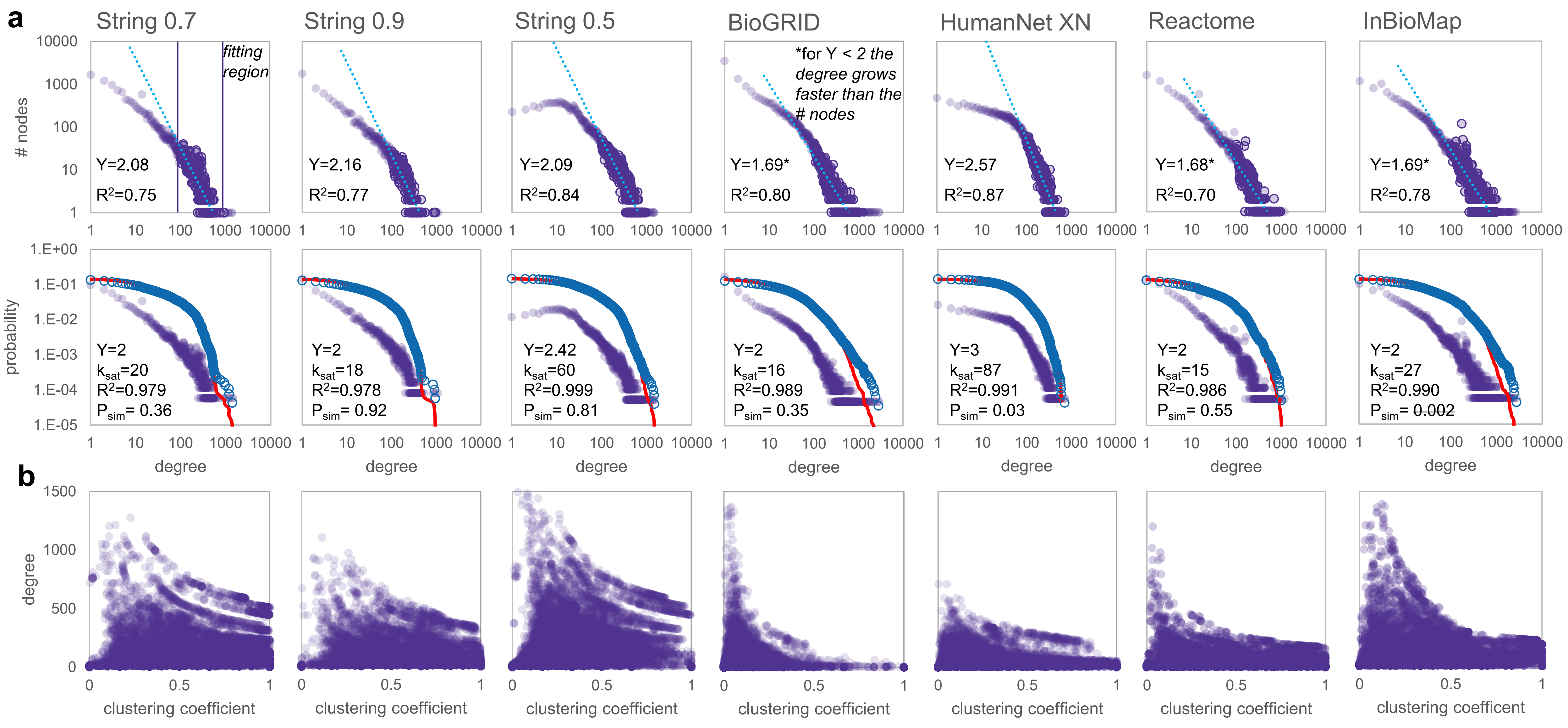

Figure S6. General features of analyzed protein functional networks. **a.** Degree distributions, fit directly to a power law function (top) and to a cumulative probability function with low degree saturation correction (bottom). In this analysis, the cumulative function fit exhibits non null probability of diverging from the raw data for the InBioMap network. **b.** Scatter plots of degree vs clustering coefficient for the same networks.

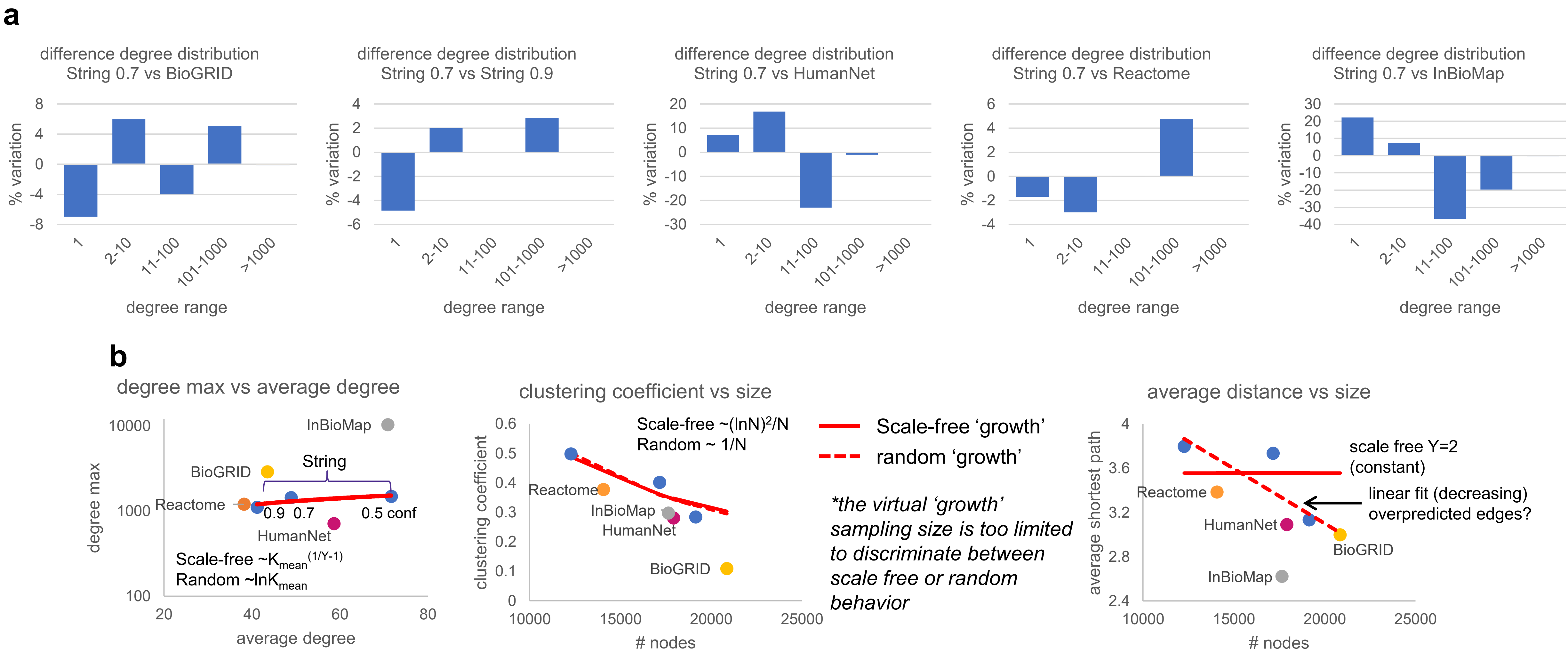

Figure S7. Comparative evaluation of analyzed protein functional networks. **a.** Relative percentile variation between networks in nodes distribution across degree ranges. **b.** network 'growth' metric plots evaluating scale-free versus random growth behavior (especially for the addition of nodes and edges to the String network at decreasing confidence level). Given the relatively small differences in network size it is not possible to discriminate between scale-free versus random characteristics of the entire String network based on its size variation after varying the confidence level. The other analyzed networks are plotted for comparison.

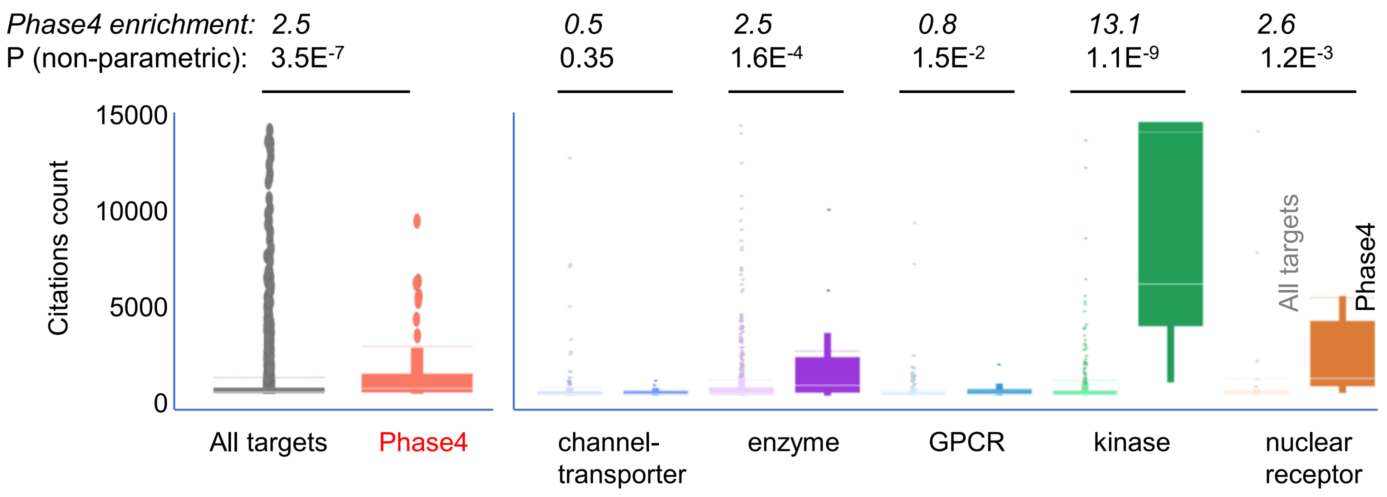

Figure S8. Box plots representing number of literature citations associated with Phase4 targets vs all targets with relative enrichment of Phase4 target citations and non-parametric significance probabilities.

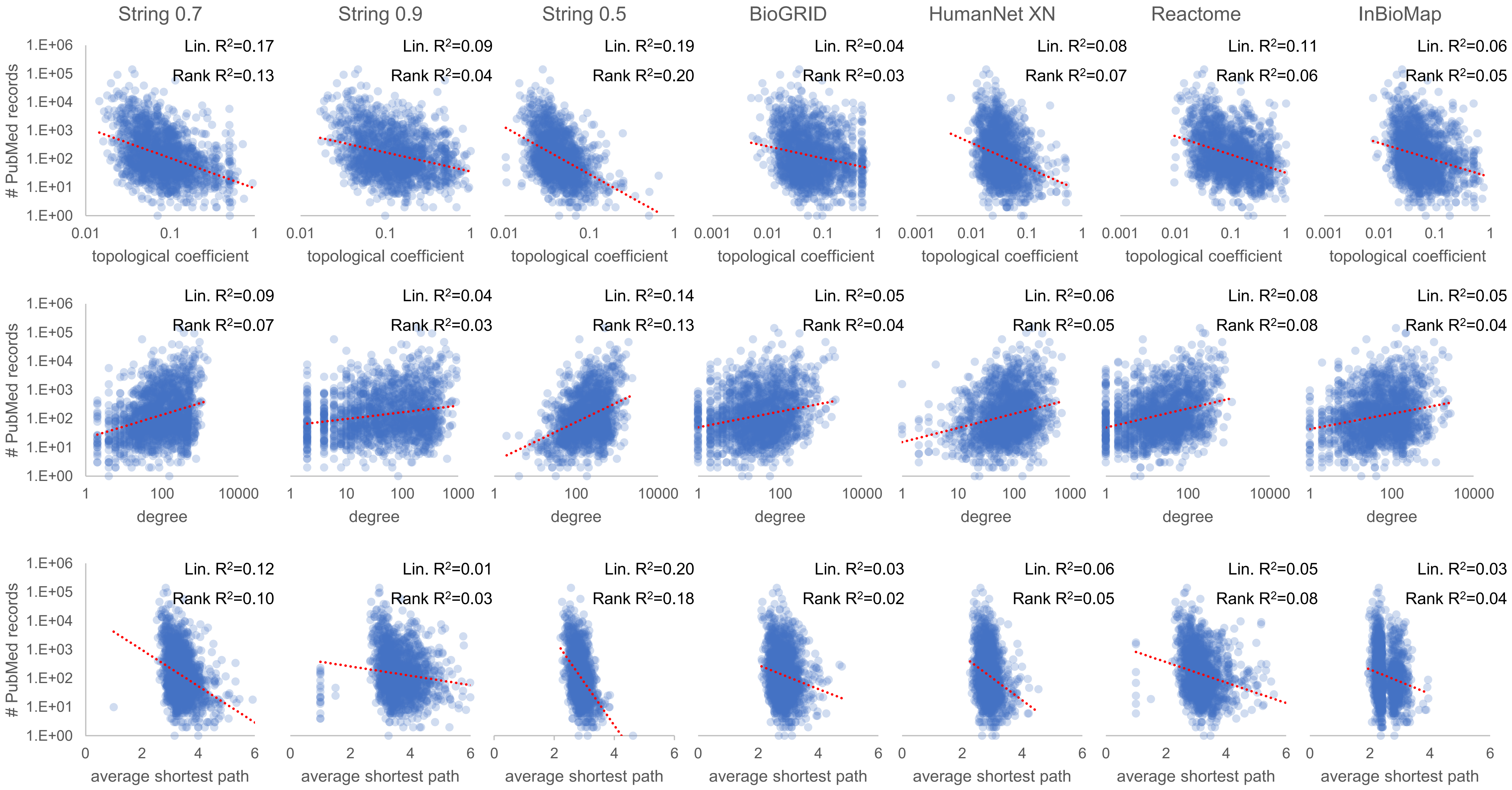

Figure S9. Linear regression and Spearman rank correlations between node parameters and number of PubMed/records of individual nodes (Phase4 and all targets merged) in the various analyzed networks.

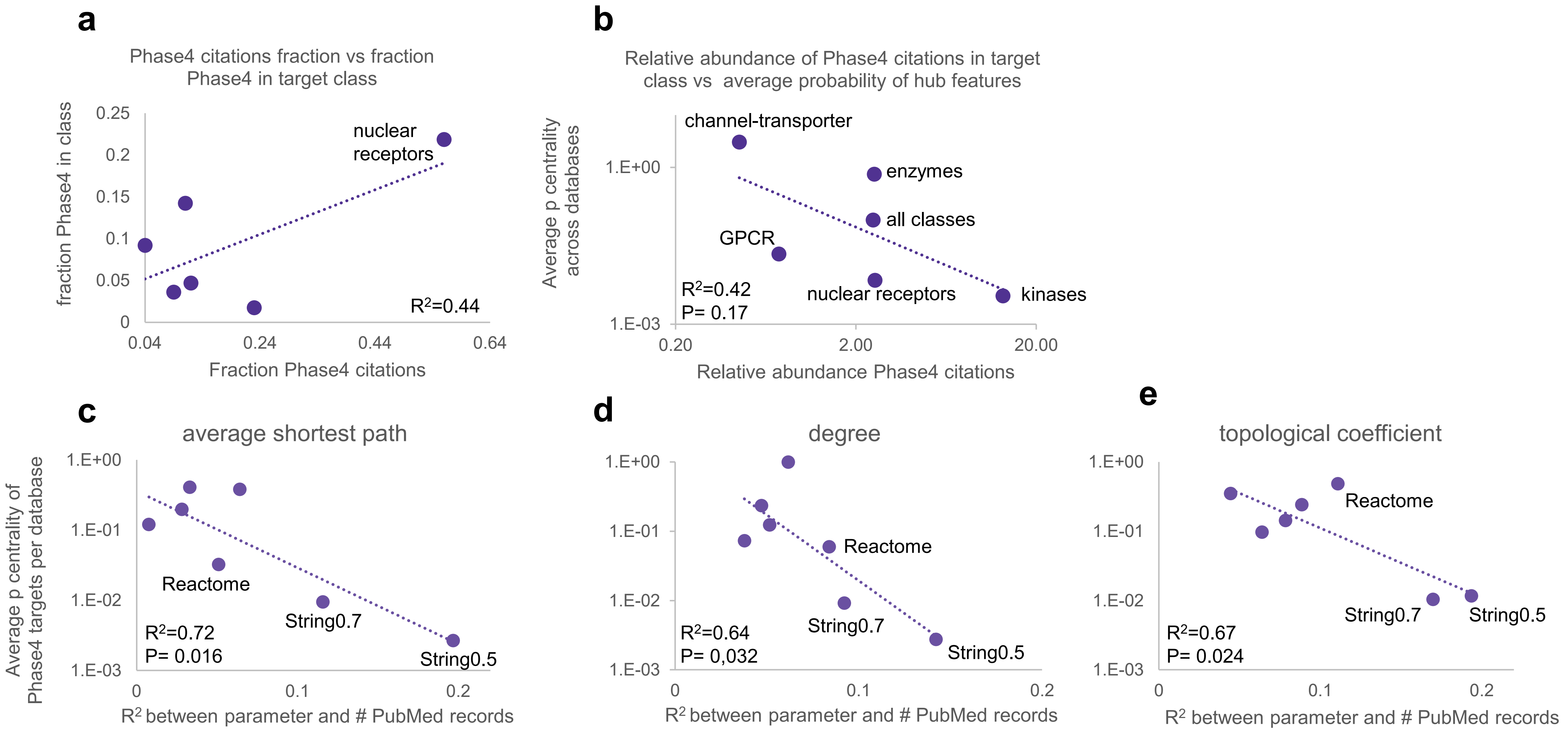

Figure S10. Inspection of correlations between protein network centrality metrics and relative enrichment in PubMed records. **a.** Scatter plots of relative number of citations for Phase4 targets versus fraction of Phase4 targets within each target class. **b.** Relative abundance of Phase4 targets citations within each target class versus the average probability of increased centrality within each target class, across networks (average non-parametric probability across networks of lower average shortest path, topological coefficient, higher degree for Phase4 over all targets, not corrected for multiple testing). **c-e.** Target-class averaged probabilities of Phase4 targets increased centrality (not corrected for multiple testing) in each network plotted against correlations between the specified centrality parameter and number of PubMed records (as displayed in Figure S9). This analysis identifies increased probability of Phase4 targets network centrality in networks where centrality parameters exhibit higher correlations with citation counts.

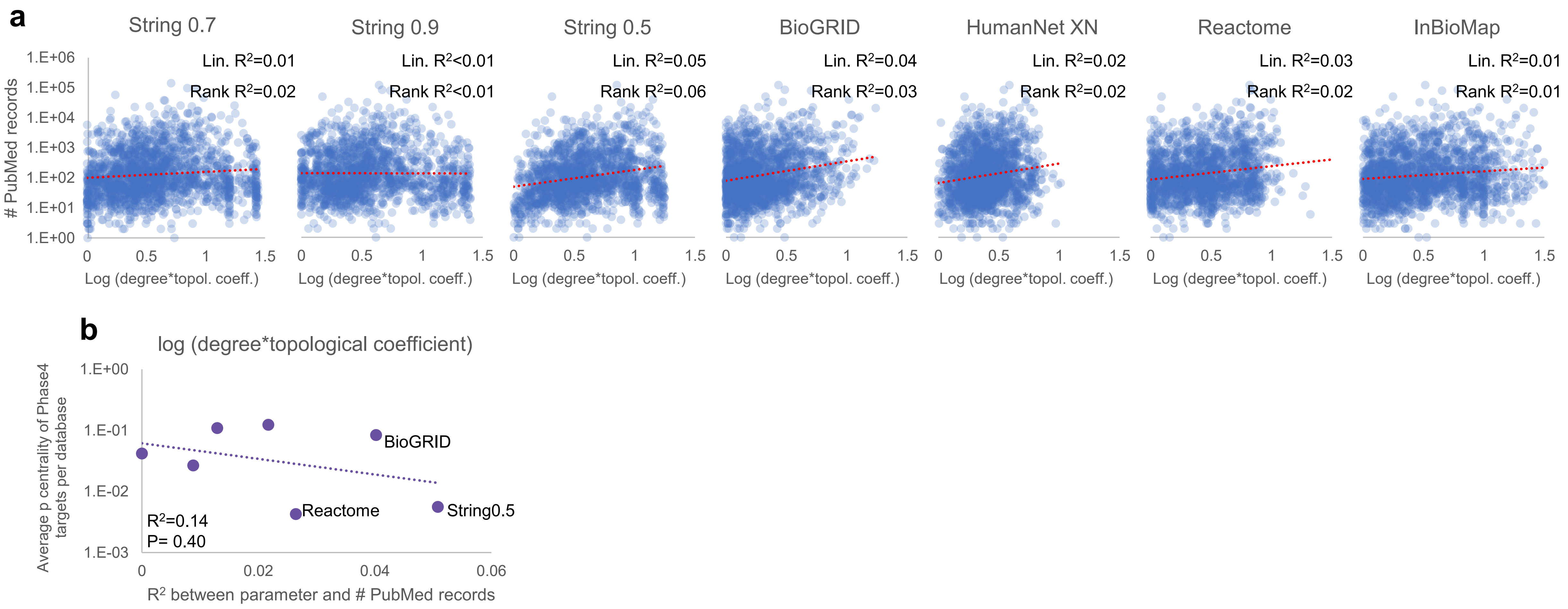

Figure S11. Linear regression and Spearman rank order correlations between the 'degree-weighted' parameter *log (degree*topological coefficient)* and number of PubMed/records of individual nodes (Phase4 and all targets) in the various analyzed networks (**a**). **b.** Target-class averaged probabilities of differences in this combined parameter between Phase4 and all targets for each analyzed network plotted against correlations between the specified centrality parameter and number of PubMed records for the corresponding node in the same database (as displayed in **a**). As the *log (degree*topological coefficient)* parameter introduces reciprocity between two centrality measures, correlations with the number of PubMed records are largely abrogated. Analysis of this parameter therefore ought to identify features of drug target nodes unbiased by their relatively enrichment in literature citations.

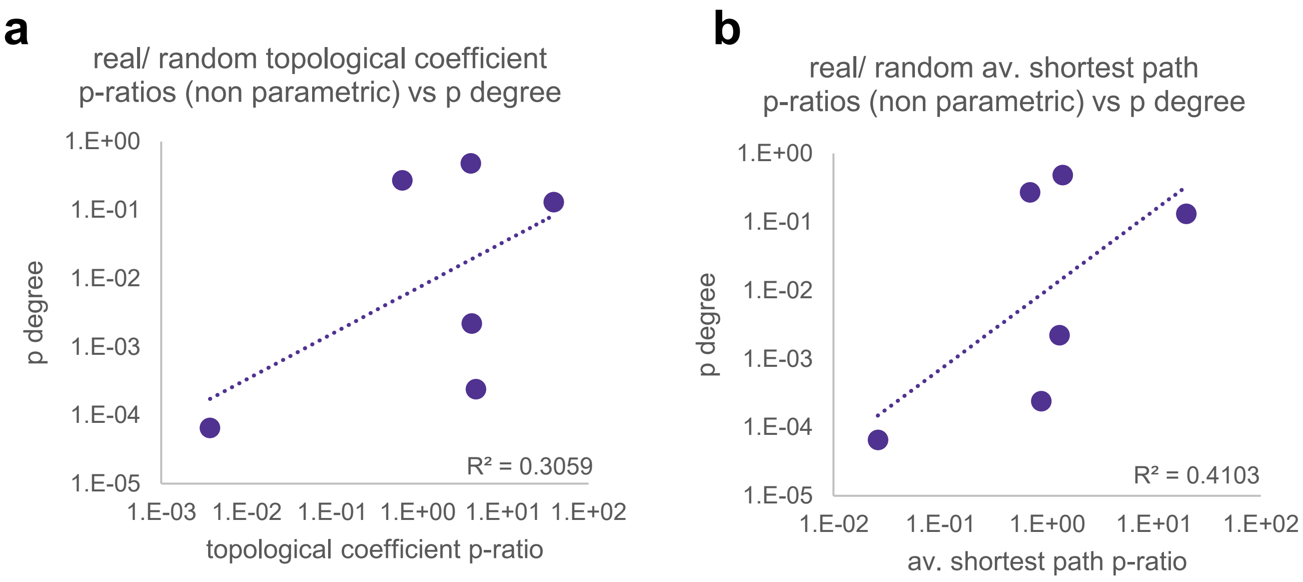

Figure S12. Scatter plots of probability of differences in degree between Phase4 targets and all targets versus variations in probabilities (not corrected for multiple testing) of difference in topological coefficient (**a**) and average shortest path (**b**) after degree-preserving randomization.

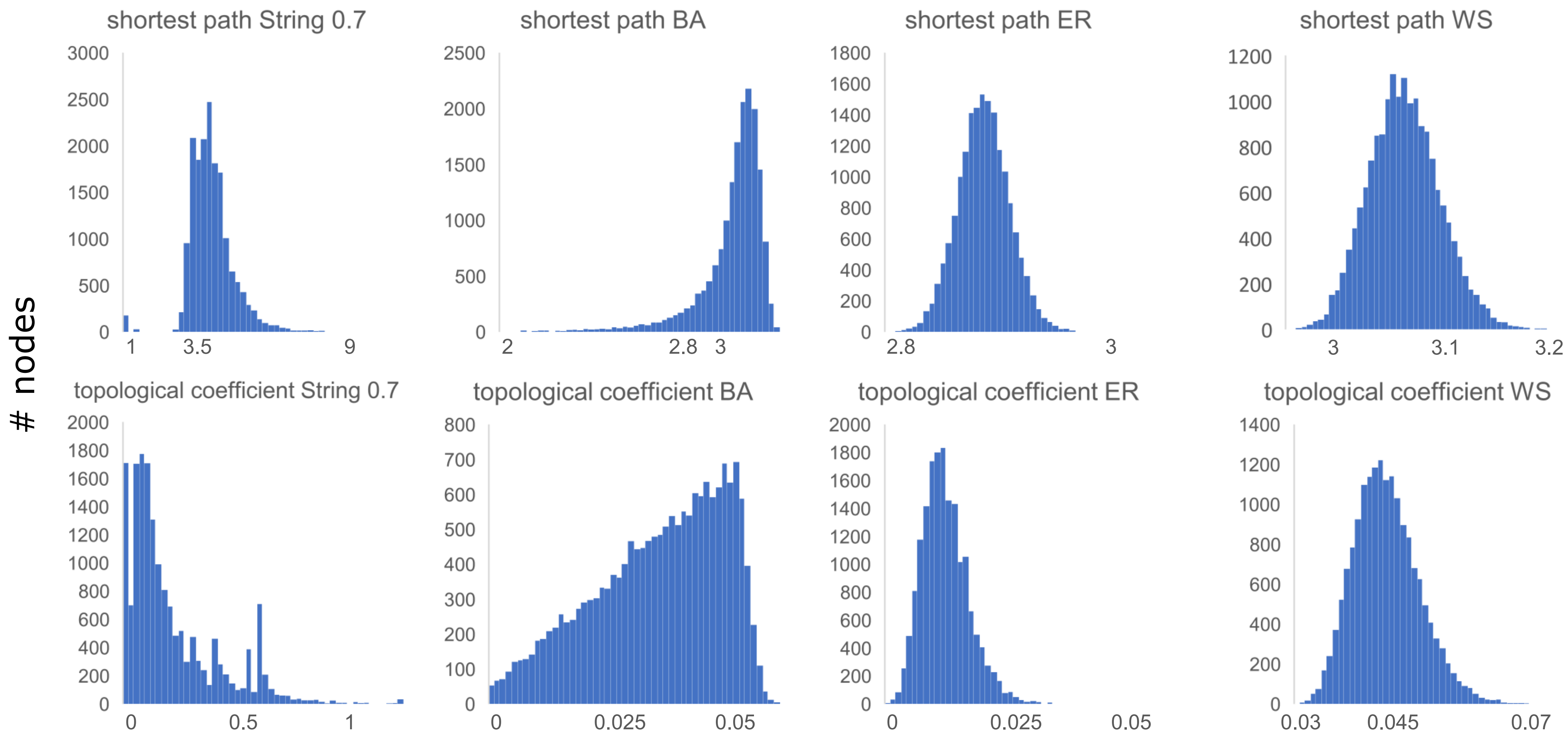

Figure S13. Distribution of centrality parameters in randomized networks.

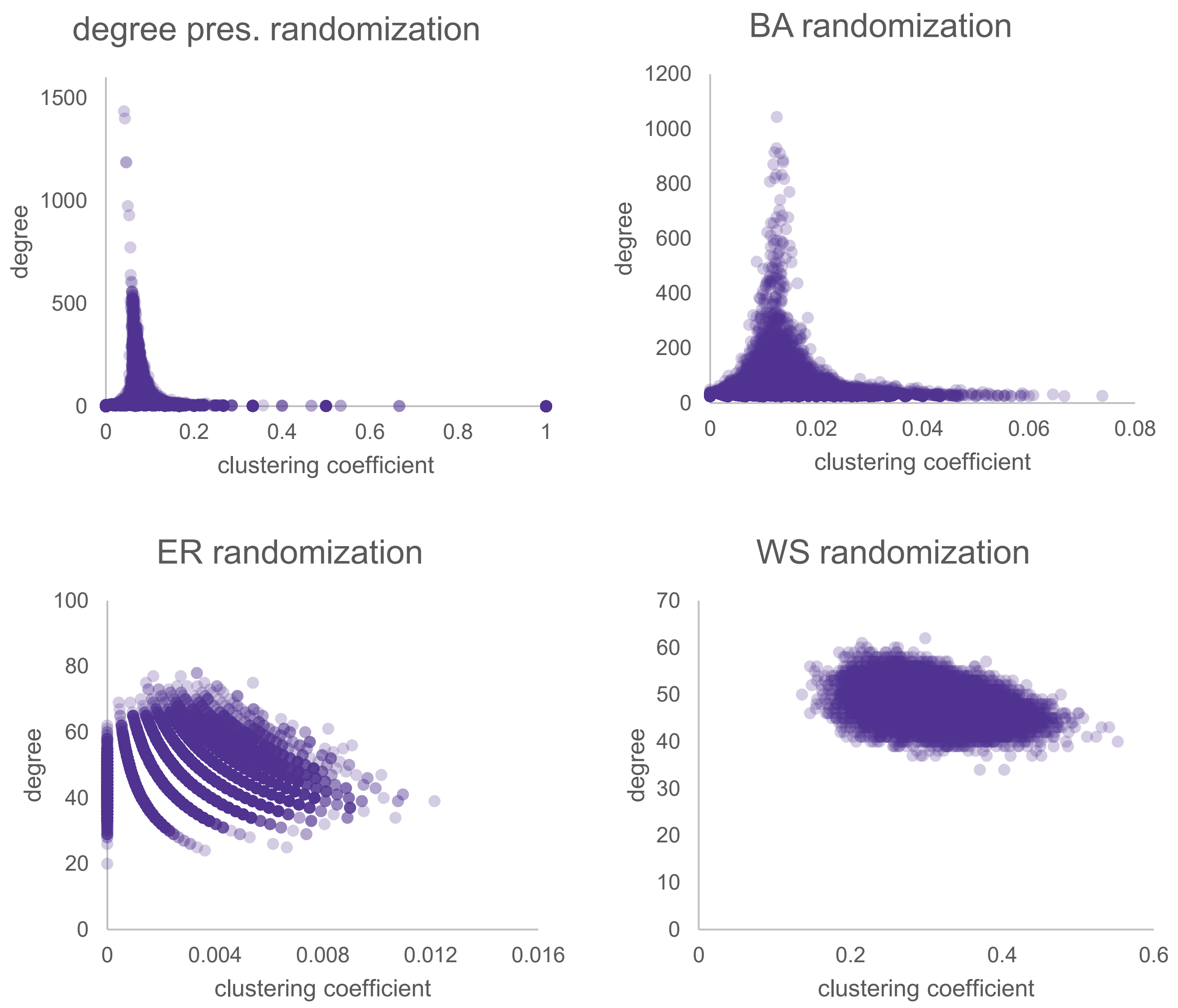

Figure S14. Plots of clustering coefficient versus degree for randomized networks of comparable size to the String0.7 network.

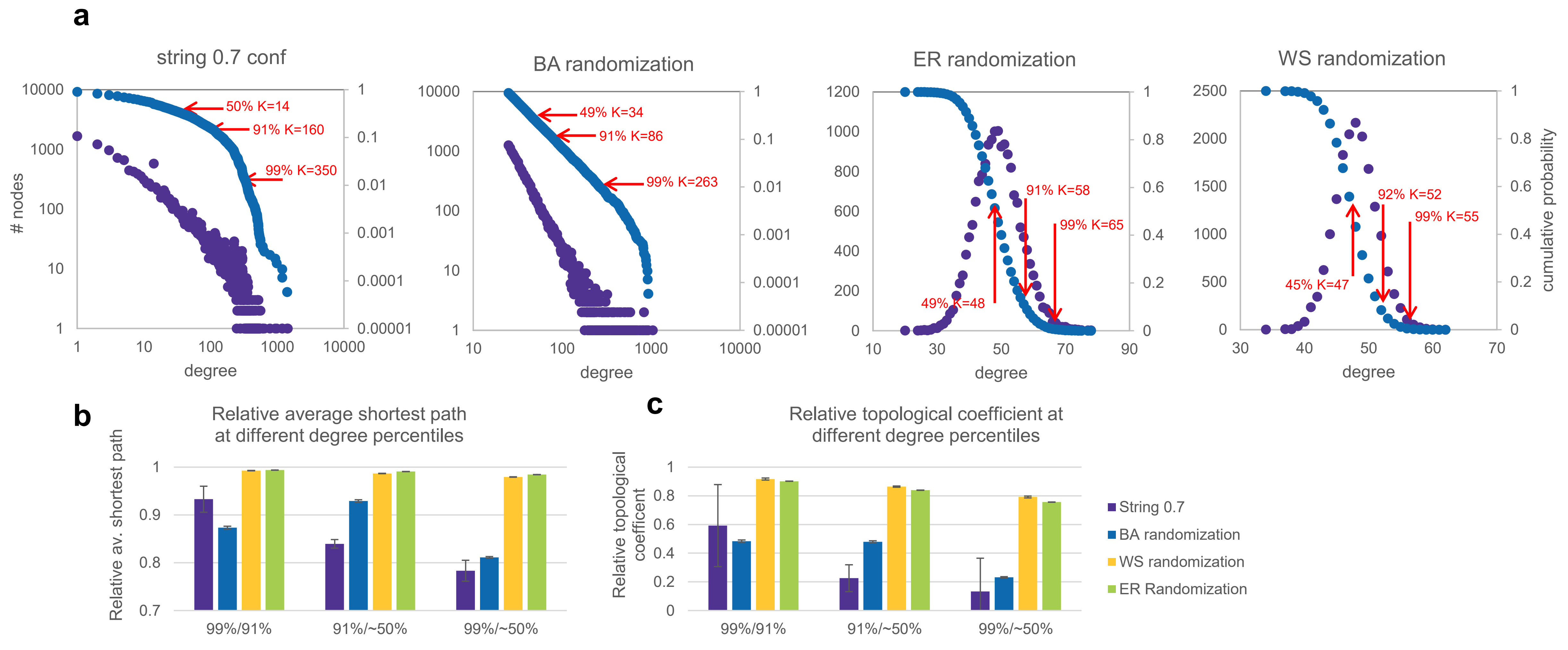

Figure S15. Varying correlations between centrality parameters in different network structures. **a.** Identification of degree values of equivalent cumulative percentile distribution (~50**%,** 91% and 99%) in different networks. **b-c.** Relative variation of average shortest path (**b**) and topological coefficient (**c**) at different degree values in different networks. Scale-free networks exhibit a larger relative variation in centrality metrics relative to random networks.

		relative K_{mean}	*relative standard deviation*
Phase4 (N = 7)	**String 0.7**	**0.653**	**0.087**
	ER-1	0.650	0.087
	ER-2	0.658	0.088
	WS-1	0.656	0.085
	WS-2	0.651	0.088
All targets (N = 32)	**String 0.7**	**0.569**	**0.131**
	ER-1	0.569	0.131
	ER-2	0.571	0.131
	WS-1	0.569	0.130
	WS-2	0.576	0.129

Table S5. Normalized degree distribution parameters of random nodes samples utilized to compare the correlations of centrality metrics between scale-free and random graphs.

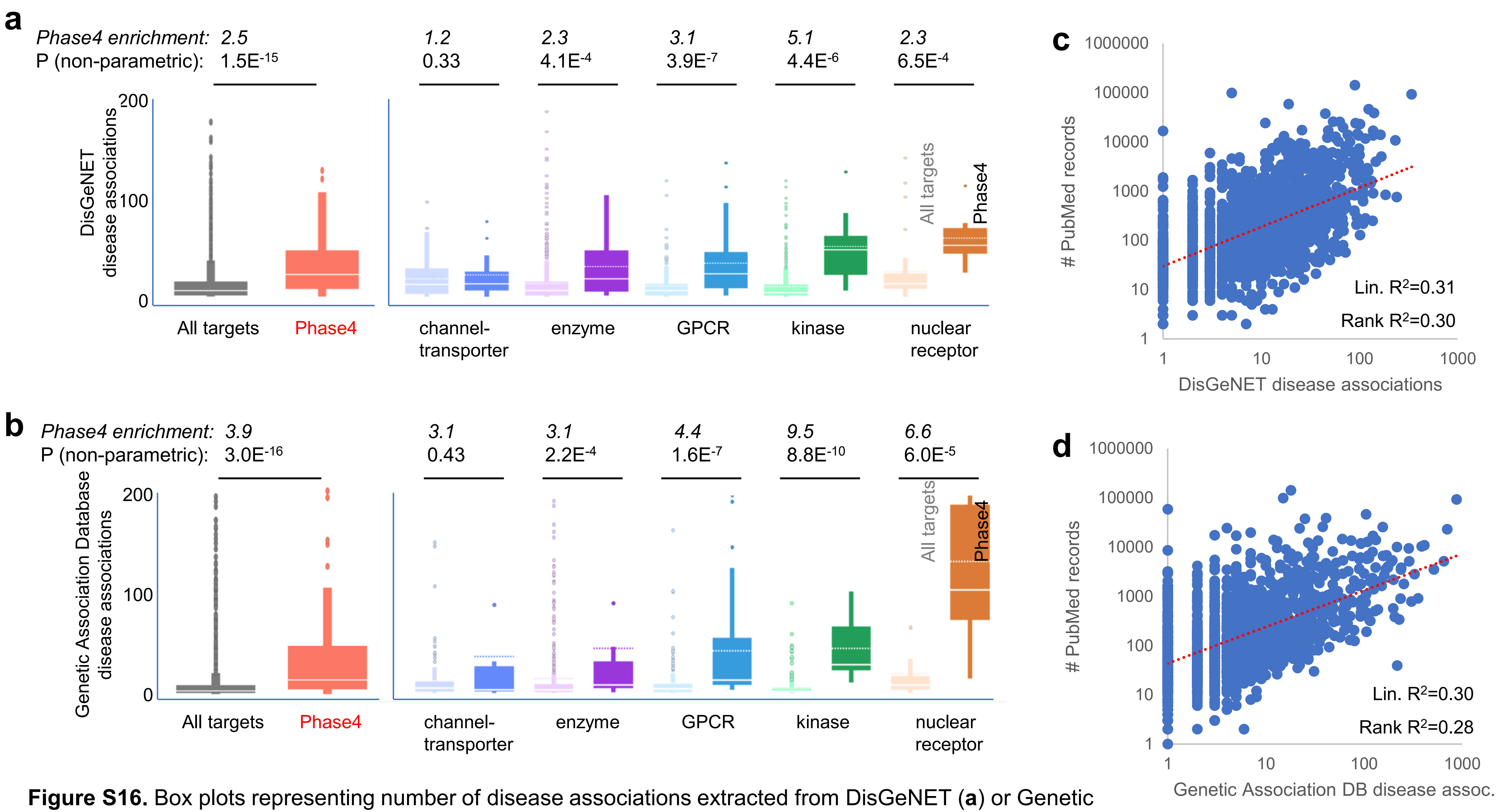

Figure S16. Box plots representing number of disease associations extracted from DisGeNET (**a**) or Genetic Association databases (**b**) with Phase4 targets vs all targets citations and non-parametric significance probabilities. **c-d.** Corresponding scatter plots highlighting positive correlations between disease association annotations and number of PubMed records for proteins in the Phase4 and all targets sets.

predictive model	naïve Bayesian model training parameters					
	**Graph + PFAM**			**Graph + PFAM + disease assoc. + GO terms**		
	default P	min. st. dev.	thrshld. st. dev.	default P	min. st. dev.	thrshld. st. dev.
String0.7 full network	1.0E-08	1.0E-04	1.0E-03	5.0E-05	9.0E-04	5.0E-04
channels-transporters	1.0E-07	1.0E-04	1.0E-03	1.3E-03	2.5E-03	5.5E-04
enzymes	1.0E-06	2.4E-04	2.4E-04	1.0E-05	5.4E-04	3.0E-03
GPCRs	1.0E-06	2.0E-02	1.0E-04	1.5E-03	3.5E-02	3.5E-02
kinases	1.0E-05	1.0E-04	1.0E-03	1.0E-04	1.0E-04	1.0E-04
nuclear receptors	1.0E-06	1.0E-03	1.0E-03	1.0E-08	3.5E-02	3.5E-02
**cumulative class models**	---	---	---	---	---	---
**with >0.99 conf. unclass.**	1.0E-08	1.0E-04	1.0E-03	5.0E-05	9.0E-04	5.0E-04
Reactome full network	1.0E-09	5.5E-04	1.0E-03	2.0E-05	1.0E-04	1.0E-04
channels-transporters	1.0E-04	8.0E-04	1.0E-03	1.0E-03	1.0E-03	5.0E-04
enzymes	1.0E-04	1.4E-04	1.0E-03	2.3E-04	1.0E-05	1.0E-05
GPCRs	1.0E-04	1.0E-03	1.0E-03	1.0E-03	1.0E-03	1.0E-03
kinases	1.0E-04	1.0E-03	1.0E-03	1.0E-03	1.0E-03	1.0E-03
nuclear receptors	1.0E-04	2.0E-03	1.0E-03	1.0E-03	1.0E-03	1.0E-03
**cumulative class models**	---	---	---	---	---	---
**with >0.9 conf. unclass.**	1.0E-09	5.5E-04	1.0E-03	2.0E-05	1.0E-04	1.0E-04

Table S6. Training parameters utilized in each naïve Bayesian predictive model shown in Tables 11 and 14.

	Disease association database		disease associations	RN score	R top	R top-degree
String07	DisGeNET	R^2 linear (*rank*)	0.31 (*0.3*)	0.13 (*0.12*)	0.16 (*0.17*)	0.16 (*0.16*)
	Genetic Association Database		0.3 (*0.28*)	0.13 (*0.11*)	0.16 (*0.17*)	0.15 (*0.16*)
Reactome	DisGeNET		0.31 (*0.3*)	0.24 (*0.2*)	0.22 (*0.2*)	0.15 (*0.12*)
	Genetic Association Database		0.31 (*0.28*)	0.25 (*0.22*)	0.25 (*0.24*)	0.19 (*0.13*)

Table S7. Correlations between number of PubMed records for proteins in the String07 and Reactome networks and number of disease associations or derived ‘hybrid’ disease association – graph centrality metrics (RN score, R top, R top-degree). Combinations of disease associations ad graph centrality metrics dilute the strong correlation observed between disease associations and number of literature references for proteins in these networks.
